# Stoichiometric balance ratio of cellobiose and gentiobiose induces cellulase production in *Talaromyces cellulolyticus*

**DOI:** 10.1186/s13068-023-02296-1

**Published:** 2023-03-16

**Authors:** Shivam Aggarwal, Sathish Dorairaj, Nidhi Adlakha

**Affiliations:** Synthetic Biology and Bioprocessing Group, Regional Centre for Biotechnology, NCR-Biotech Cluster, Faridabad, India

**Keywords:** Cellulase, Inducer, Cellobiose, *Talaromyces* sp., Gentiobiose

## Abstract

**Background:**

The exact mechanism by which fungal strains sense insoluble cellulose is unknown, but research points to the importance of transglycosylation products generated by fungi during cellulose breakdown. Here, we used multi-omics approach to identify the transglycosylation metabolites and determine their function in cellulase induction in a model strain, *Talaromyces cellulolyticus* MTCC25456.

**Results:**

*Talaromyces* sp. is a novel hypercellulolytic fungal strain. Based on genome scrutiny and biochemical analysis, we predicted the presence of cellulases on the surface of its spores. We performed metabolome analysis to show that these membrane-bound cellulases act on polysaccharides to form a mixture of disaccharides and their transglycosylated derivatives. Inevitably, a high correlation existed between metabolite data and the KEGG enrichment analysis of differentially expressed genes in the carbohydrate metabolic pathway. Analysis of the contribution of the transglycosylation product mixtures to cellulase induction revealed a 57% increase in total cellulase. Further research into the metabolites, using in vitro induction tests and response surface methodology, revealed that *Talaromyces* sp. produces cell wall-breaking enzymes in response to cellobiose and gentiobiose as a stimulant. Precisely, a 2.5:1 stoichiometric ratio of cellobiose to gentiobiose led to a 2.4-fold increase in cellulase synthesis. The application of the optimized inducers in cre knockout strain significantly increased the enzyme output.

**Conclusion:**

This is the first study on the objective evaluation and enhancement of cellulase production using optimized inducers. Inducer identification and genetic engineering boosted the cellulase production in the cellulolytic fungus *Talaromyces* sp.

**Supplementary Information:**

The online version contains supplementary material available at 10.1186/s13068-023-02296-1.

## Introduction

Soil-borne fungi can mediate plant biomass degradation [1, 2]. These fungi produce hydrolytic enzymes, which have been explored extensively for their use in biorefineries [3–7]. Despite the available scientific knowledge on enzyme-based deconstruction of biomass, development of efficient enzyme cocktail for cost-effective release of fermentable sugars for biorefineries is still a major challenge [8, 9]. The genomes of these saprophytic fungi encode for a wide range of cellulolytic enzymes broadly categorized under Carbohydrate-Active enZymes (CAZymes). These are majorly grouped into five classes, namely, glycoside hydrolases (GHs), carbohydrate esterases (CEs), polysaccharide lyases (PLs), glycosyl transferases (GTs), and auxiliary activities (AAs) in the CAZy database [10–15]. Among them, GHs, CEs, and PLs play an important role in plant cell wall degradation and are commonly referred to as cell wall degrading enzymes (CWDEs). The efficient degradation of complex lignocellulose into its monomers is highly dependent on the expression of these inducible CWDEs by the saprophytic fungi [16].

*Trichoderma reesei* is considered as powerhouse for production of cellulolytic enzymes at an industrial scale and has been widely studied for cellulase production. Over the decades, genetic engineering approaches have been employed to increase the cellulolytic potential of the fungus. CreA is a transcription factor that plays a major role in carbon catabolite repression (CCR) in cellulolytic fungi and has been shown to represses the expression of various cellulase genes [17]. *T. reesei* RUT C-30 contains a truncated *creA* and is therefore CCR free [17–19]. This strain is widely used for industrial cellulase production.

However, recent genomic and transcriptomic studies have shown that this fungus possess poor diversity of hydrolytic enzymes than other known cellulose-degrading fungal species and therefore cellulase cocktails developed from *T. reesei* require an additional supplementation with accessory hydrolases to achieve complete disintegration of the recalcitrant cellulose [20, 21]. Besides, recent reports have suggested that enzyme preparation from different fungi outperform *T. reesei*-based enzymatic cocktail [22, 23], accentuating the need to explore for alternatives.

Along with identifying a hypercellulolytic fungus strain, there is a need to comprehend the process underlying cellulase secretion to meet industrial demand for enzymes. Cellulases being adaptive enzymes, their expression requires the presence of a soluble inducer. Although complex cellulose is an acknowledged inducer of cellulase biosynthesis, it cannot enter into the cell to initiate the induction cascade. Kubicek et al., 2009 suggested that the constitutive cellulases act on insoluble cellulose and release saccharides which induce cellulase production [24]. While various cellulose-derived oligosaccharides, such as cellobiose, lactose, maltose, and sophorose, have been previously explored for their role as inducers, which showed low to moderate induction levels [25–28], the rational scrutiny of oligosaccharide inducers for cellulase production is still missing.

The present study aimed to unravel the cellulase inducers in hypercellulolytic strain using multi-omics approach. In the current study, we have identified a novel fungal strain, *Talaromyces cellulolyticus*, which outperformed industrial strains, such as *T. reesei* and *A. niger*, in terms of total protein and enzyme production. First, we sequenced the 35.6 Mb whole genome of the *Talaromyces cellulolyticus* encoding 11,032 proteins, of which more than 600 CDS encoded for CAZymes. Transcriptomic and metabolomic analyses were performed to reveal the early metabolites and their role in cellulase induction. The ideal inducer combination identified using Plackett–Burman statistical design significantly enhanced the cellulase induction. Additionally, we evaluated the effect of optimal inducer in the ΔcreA strain and observed remarkably improved biomass saccharification. The knowledge of specific inducers will certainly revolutionize the cellulase industry.

## Results

### Genome sequencing and analysis of the transcriptional responses of *Talaromyces* sp. to polysaccharide substrate

**Fig. 1:**
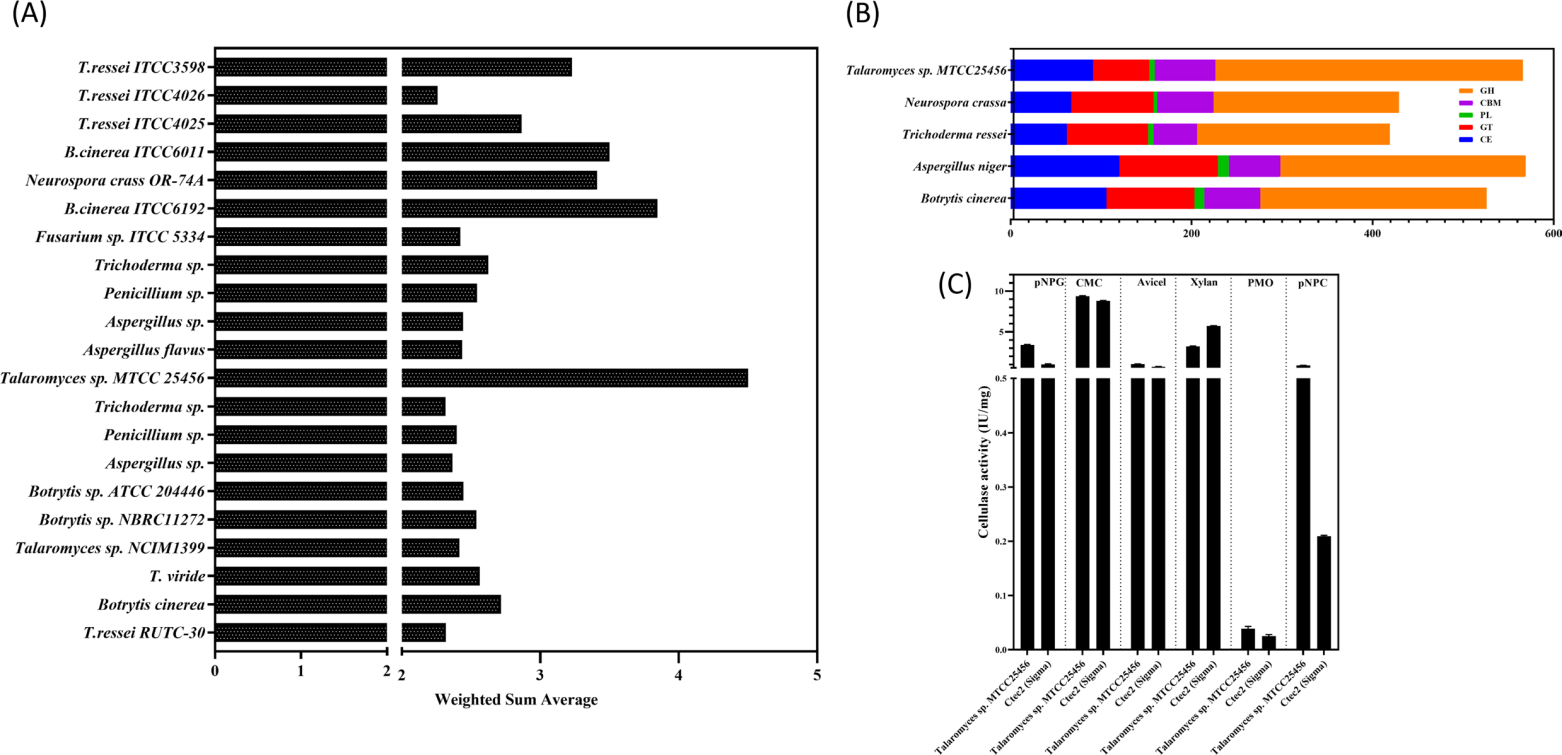
Comparative CAZyme distribution in *Talaromyces* sp. MTCC25456. **a** WSM for different cellulolytic fungi. **b** Comparative analysis of CAZymes suggested *Talaromyces* sp. genome harbors 649 CDS encoding for proteins involved in carbohydrate disintegration. **c** Specific activities for various components of biomass disintegrating enzymes in *Talaromyces* sp. and its comparison with commercial enzyme cocktail

We screened the soil samples for industrially relevant cellulose-degrading fungal species and compared their biomass saccharification capability with that of recognized biomass-hydrolyzing fungi procured from collection centers, like ATCC, ITCC, and NCIM. To assess their cellulolytic capacity, we conducted biochemical assays, like avicelase, CMCase, and pNPGase, in their culture supernatants (Additional File 1: Fig. S1). Based on Gao et al. study, the contribution of core cellulases to polysaccharide hydrolysis was analyzed and we developed a weighted cumulative evaluation model [29, 30]. The model demonstrated that *Talaromyces cellulolyticus* dominated the weighted sum score comparison and therefore was chosen for further investigation (Fig. 1a).

The phylogenetic analysis was performed based on two established molecular markers for fungal identification: interspace transcribed sequences (ITS2) and beta-tubulin (BenA). The ITS2-based phylogenetic tree predicted its co-relation to the clade which contain species, like Pezizomycetes, Harmoniella, *C. podzolicus*, Tremellodendropsis, *F. aurantiaca*, *E. tinctorium*, *B. roseogriseus*, Boletus, Aureoboletus, Chalciporus, and Phylloporus, while being closely related to other *Talaromyces* sp. and *Aspergillus* sp. (Additional File 1: Fig. S2A). On the other hand, beta-tubulin-based phylogenetic tree predicted that the isolate is closely related to *Talaromyces cellulolyticus* and *Talaromyces marneffei* ATCC 18224 (Additional File 1: Fig. S2B). Therefore, based on cumulative analysis, we annotated the fungal isolate as *Talaromyces cellulolyticus* fungus. The strain has been deposited in Microbial Type Culture Collection, Chandigarh with accession number MTCC 25456.

To investigate the polysaccharide disintegrating ability of the strain, we sequenced the genome of *Talaromyces* sp. using Illumina HiSeq 2500 platform, and a total of 31,673,084 bp paired-end reads were obtained which assembled into 828 contigs. The cleaned reads were subjected to Kmergenie [31] to predict the optimal k-value and assembly size that was found to be 78 and 38,535,829 bp, respectively. Based on its closest homolog, *Talaromyces pinophilus*, the synteny plot of assembled contigs of *Talaromyces* sp. was created (Additional File 1: Fig. S3), which suggested that *T. cellulolyticus* MTCC25456 genome consisted of eight chromosomes.

The data were assembled using several assemblers, namely, SOAPdenovo [32], velvet [33], MaSuRCa [34], and Abyss [35] (Additional File 1: Table S1), with multiple kmer values to select the best assembly. The qualitative and quantitative analyses of the assemblies have been performed using BUSCO [36] and Quast [37], respectively. The assembly built by the MaSuRCa-3.2.2 has been identified to be the best and selected for the downstream analysis (GenBank assembly accession number GCA_009805475.2). We predicted CDSs from the MaSuRCa assembled contigs using Augustus version 3.2.3 and found 11,032 predicted genes. Gene ontology study catalogued the genes into 627 molecular function, 424 biological process, and 247 cellular component category (Additional File 1: Fig. S4). KEGG analysis assigned predicted genes to 489 pathways, wherein 208 pathways belonged to secondary metabolite biosynthesis.

Numerous reports suggest that the cell wall degrading enzymes are adaptive, and thus, its secretion by fungi is driven by the presence of polysaccharide substrate [3–5, 38]. We analyzed perturbation in transcriptome in response to polymeric substrate to conceive the biomass hydrolytic capability of newly isolated strain. A number of differentially expressed genes (FDR < 0.05, |log2FC|> 0.9, p < 0.05) were observed in the presence of polysaccharide substrate wrt glucose (Sequence Read Archive submission: SUB8465489). Expectedly, RNA sequence analysis of *Talaromyces* sp. suggested predominance of biomass-hydrolyzing enzymes. The data suggested secretion of a range of CAZymes, including Glycosyl hydrolases, such as endo-β-1,4-glucanase (GH5), reducing-end acting cellobiohydrolase (GH7), endoxylanase (GH12), and oligoxyloglucan reducing-end-specific cellobiohydrolase (GH74), and accessory biomass disintegrating enzymes, such as lytic polysaccharide monooxygenase (AA9), cellobiose dehydrogenase (AA3), and swollenin (Additional File 1: Fig. S5B).

### Biomass-degrading enzymes in *Talaromyces cellulolyticus*

The biomass-degrading enzymes are categorized by dbCAN (DataBase for automated Carbohydrate-active enzyme ANnotation) into different classes and families based on substrate specificity and cleavage site [39]. The cocktail of CAZymes secreted by any fungal species is central to its potential to degrade different types of polysaccharide substrate and therefore crucial in designing optimal enzyme mixture for complete depolymerization of biomass into monomeric fermentable sugars. In light of this, we performed a cumulative CAZome analysis of *Talaromyces* sp. and compared it with other hypercellulolytic fungal strains. For genome-wide comparisons, amino acid sequences of 4 fungal species were analyzed using the HMMER 3.0 package (http://hmmer.org/) and dbCAN CAZyme database (http://csbl.bmb.uga.edu/dbCAN/). The results show that *Talaromyces* sp. harbors exceptionally higher numbers of CAZymes [649], suggesting that the strain has an unmatchable tendency to degrade complex biomass (Fig. 1b). The total putative CWDE proteins were classified into 67 CBM, 340 GH, 91 CE, 6 PL, 62 GT, and 83 AA families. This was in accordance with the biochemical assays, which suggested that *Talaromyces* sp. secretes copious amounts of biomass depolymerizing enzymes specifically in response to induction with polysaccharide substrate (Fig. 1c).

Comprehensive understanding of CAZymes in *Talaromyces* sp. indicated prominence of GT2 class of transporters with 12 genes (Additional File 1: Fig. S5B), which function as cellulose synthase, chitin synthase, mannosyltransferase, and rhamnosyltransferase. The finding was in agreement with a recent literature indicating abundance of GT2 in other filamentous fungi [40]. Additionally, it was observed that *Talaromyces* sp. harbors diverse cellulolytic enzymes and the most prominent ones include cellulase (GH3,-5,-13,-18,-31,-43,-78), xylanase (GH11,-30), and chitinase (GH18). These set of enzymes are known to exert synergistic action on polysaccharide biomass [41–44]. Another important feature was dominance of AA7 category of enzymes, an auxiliary activity enzyme involved in glucooligosaccharide oxidase, which oxidatively cleaves glucooligosaccharides into cellobiose or monomeric glucose.

### Scrutiny of early metabolites and its role in cellulase induction

*Talaromyces cellulolyticus* transcribes a baseline level of CAZymes on the outer surface of the spores (Additional File 1: Table S2). The membrane protein extract also suggested the presence of endoglucanase and glucosidase-type activity (Additional File 1: Fig. S6). The data were in accordance with study performed by Kubicek et al. , which predicted the presence of endoglucanase, glucosidase, and cellobiohydrolases on the surface of conidia. El-Gogary et al. (1989) also demonstrated basal levels of cellulases in uninduced fungi. This set of enzymes is believed to be the first wave of attack on polysaccharide substrate [45, 46].

**Fig. 2:**
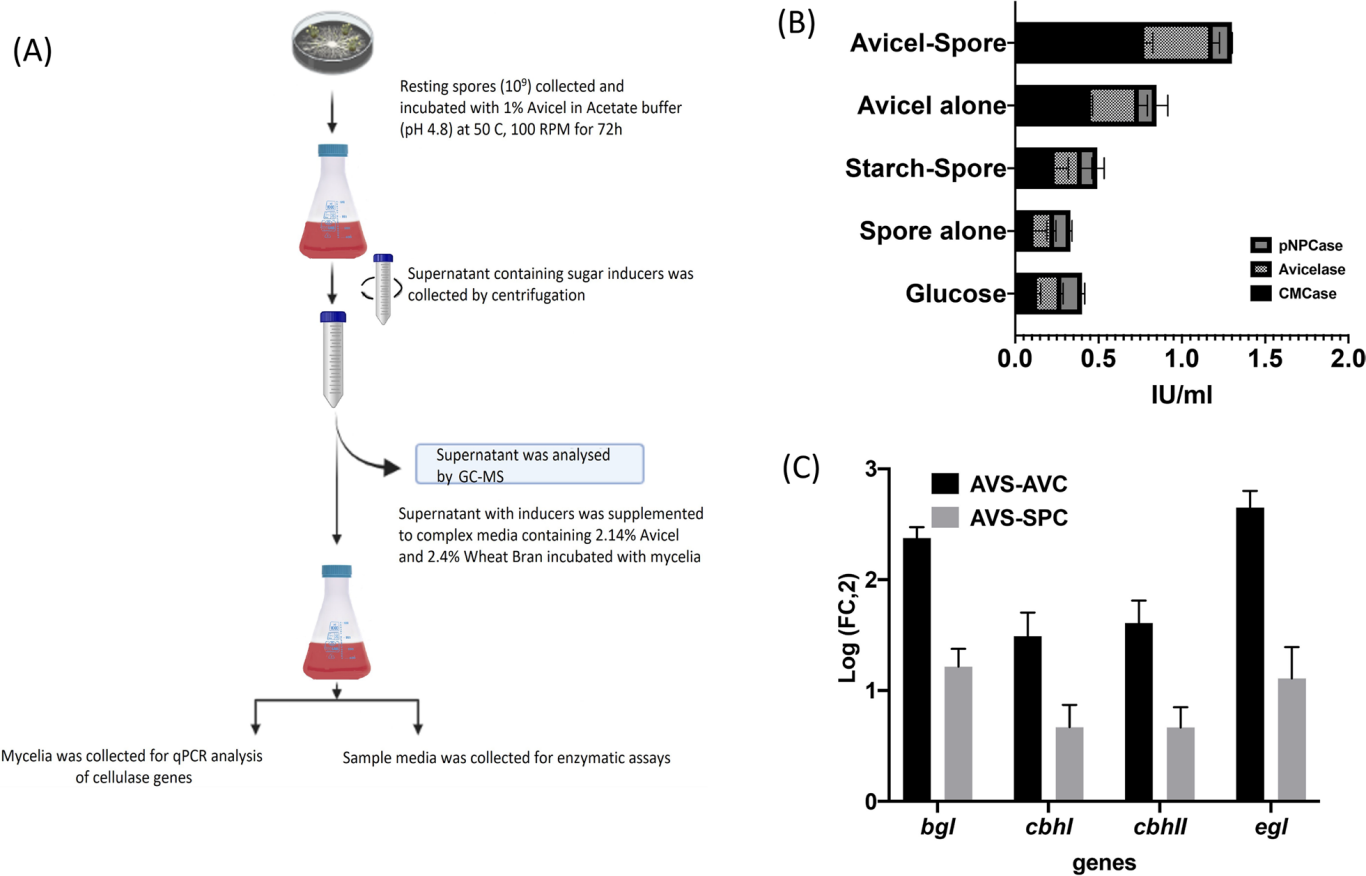
Inducer generation upon co-incubation of spores and avicel. **a** A schematic representation of experimental setup for production of potential inducer metabolite cocktail. **b** The transglycosylated metabolites, produced by co-incubation of 1% avicel with 1 × 10^9^ spores, were supplemented to liquid culture for evaluating its role as potential inducers. The culture supernatant was analyzed for its effect on cellulolytic activity, whereas metabolites produced from starch spore, spore alone, and avicel alone incubations were used as control. **c** Effect of inducers on expression of key cellulolytic gene was performed where AVS, AVC, and SPC represent avicel and spore co-incubation, avicel alone, and spore alone, respectively. Briefly, AVS, AVC, and SPC are incubated at 50 °C for 72 h and the supernatant was utilized as inducer for its effect on growth and RT-PCR analysis

The involvement of these membrane proteins in the synthesis of soluble inducer saccharides was hypothesized in the literature [47, 48]. However, rational scrutiny of inducers is still missing. Therefore, we planned a series of experiments to identify the stoichiometric ratio of inducer molecules for our model strain, *Talaromyces* sp. First, we incubated the resting spores (1X10^9^ spores/ml) with 1% avicel and the early metabolites generated by the action of spore surface enzymes were recovered in the supernatant by centrifugation. The reaction was performed at 50 °C (optimum temperature for cellulase activity) for 72 h (Additional File 1: Fig. S7). The supernatants from spore only, avicel only, and spore incubated with starch reactions were used as negative control (Fig. 2a).

The direct effect of recovered metabolite mixture was tested through a supplement experiment. Analysis of the contribution of transglycosylation product mixtures to cellulase induction revealed a 57% increase in total cellulase as compared to the controls (Fig. 2b). In concordance, it also led to significant upregulation in the transcript levels of core cellulase enzymes, i.e., cellobiohydrolase I (*cbh1*), cellobiohydrolase II (*cbh2*), beta-glucosidase-1 (*bgl1*), and endoglucanase 1 (*egl1*) (Fig. 2c; Additional File 1: Table S4). Overall, the transglycosylated products generated by the action of spore surface enzymes on avicel facilitate secretion of cellulolytic enzymes by the filamentous fungi.

We furthered our study to scrutinize the saccharides present in the mixture. For this, the supernatant was derivatized using MSTFA and subjected to GC–MS. A total of 180 metabolites were obtained in all samples. Metabolite profiles were analyzed, and chromatographic peaks detected in the spore control and avicel control were deducted from that in the test sample. The unique metabolites in the Avicel-Spore test sample (72 h) were identified by comparing them with the control (0 h). The metabolome analysis indicated the abundance of few metabolites in the supernatant (Table1). Expectedly, GC–MS results showed that the soluble sugars were the primary differential metabolites between avicel-treated and control samples.

**Table 1:** GC–MS predicted saccharides obtained by the action of spore surface enzyme on cellulose-based polysaccharide

S. no.	Name	RT	%Area
1	Glycerol, 3TMS derivative	11.546	0.59409242
2	d-(+)-Arabitol, 5TMS derivative	22.093	0.04950682
3	Arabinofuranose, 1,2,3,5-tetrakis-O-(trimethylsilyl)	21.343	0.0924836
4	d-Glucose, 2,3,4,5,6-pentakis-O-(trimethylsilyl)-, o-methyloxime, (1Z)-	26.031	26.4821871
5	d-Mannitol, 6TMS derivative	26.695	0.60446377
6	Palmitic Acid, TMS derivative	28.952	0.66104906
7	d-Lactose, octakis(trimethylsilyl) ether, methyloxime (isomer 1)	40.073	1.15422628
8	d-(+)-Cellobiose, (isomer 2), 8TMS derivative	40.116	0.08397968
9	d-Fructose, 1,3,4,5,6-pentakis-O-(trimethylsilyl)-, O-methyloxime	25.544	0.04597806
10	Talose, 5TMS derivative	26.566	12.8461313
11	Galactopyranose, 5TMS derivative	27.733	3.73120019
12	Maltose, octakis(trimethylsilyl) ether, methyloxime (isomer 1)	40.788	0.23371912
13	d-(–)-Ribofuranose, tetrakis(trimethylsilyl) ether (isomer 2)	23.405	0.97909568
14	Xylitol, 5TMS derivative	22.37	0.04147822
15	Methyl alpha-d-glucofuranoside, 4TMS derivative	24.084	0.59350895
16	d-Glucitol, 6TMS derivative	26.687	0.31814176
17	Acetin, bis-1,3-trimethylsilyl ether	9.024	0.02352991
18	beta-Lyxopyranose, 4TMS derivative	27.194	0.06650076
19	2-Alpha-Mannobiose, octakis(trimethylsilyl) ether (isomer 2)	40.523	0.15572557
20	d-Lactitol, nonakis(trimethylsilyl) ether	41.611	0.12774266
21	Beta-gentiobiose, octakis(trimethylsilyl) ether, methyloxime (isomer 1)	41.581	0.08456423

**Fig. 3:**
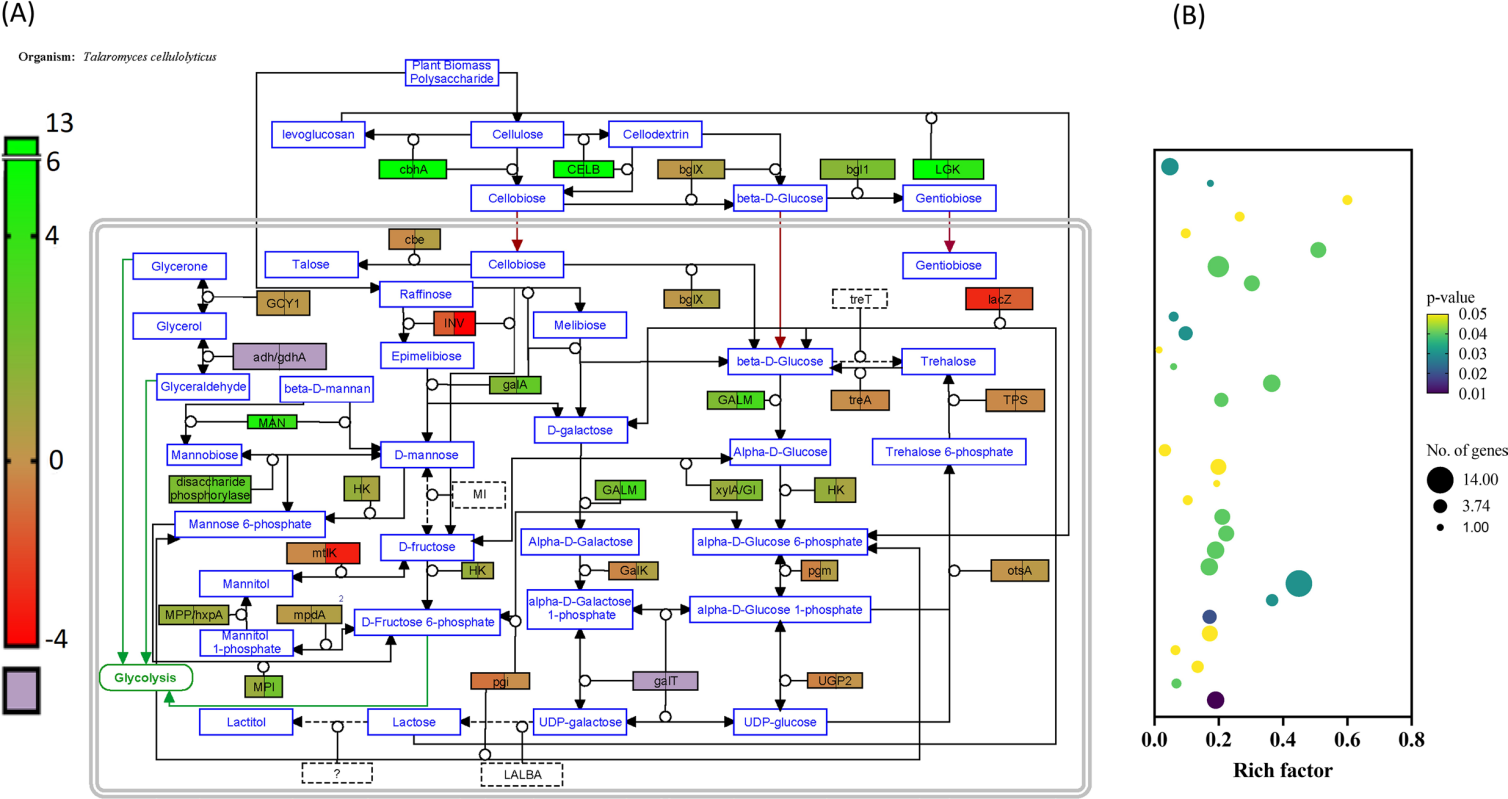
Correlation of metabolome and transcriptome in cellulase induction. **a** The transcriptome data are indicated in black bordered box, which represents differentially regulated genes in the presence of Avicel (left) or pretreated biomass (right) wrt glycerol. The purple color indicates that the genes are present in the organism, but their expression levels could not be identified. **b** KEGG Enrichment analysis. DEGs in possible pathways related with carbohydrate metabolism. The size of the solid circles represent the number of transcripts involved in the certain pathway of the rich factor

Furthermore, RNA sequencing predicted that the metabolic pathways involved in putative inducer synthesis were significantly upregulated in *Talaromyces* sp. (Fig. 3a). KEGG pathway enrichment analysis of DEGs showed maximum differential expression in the carbohydrate metabolism pathway. Compared with glucose, eight significantly enriched pathways were uniquely observed in the presence of avicel, which were “Sucrose and starch metabolism,” “TCA cycle,” “glycolysis/gluconeogenesis,” “Fructose and mannose metabolism,” “galactose metabolism,” “butanoate metabolism,” “propanoate metabolism,” and “pentose phosphate pathway metabolism” (Fig. 3b). All the eight abovementioned pathways might be correlated with the cellulase induction mechanism.

### In vitro induction studies to screen inducer

**Fig. 4:**
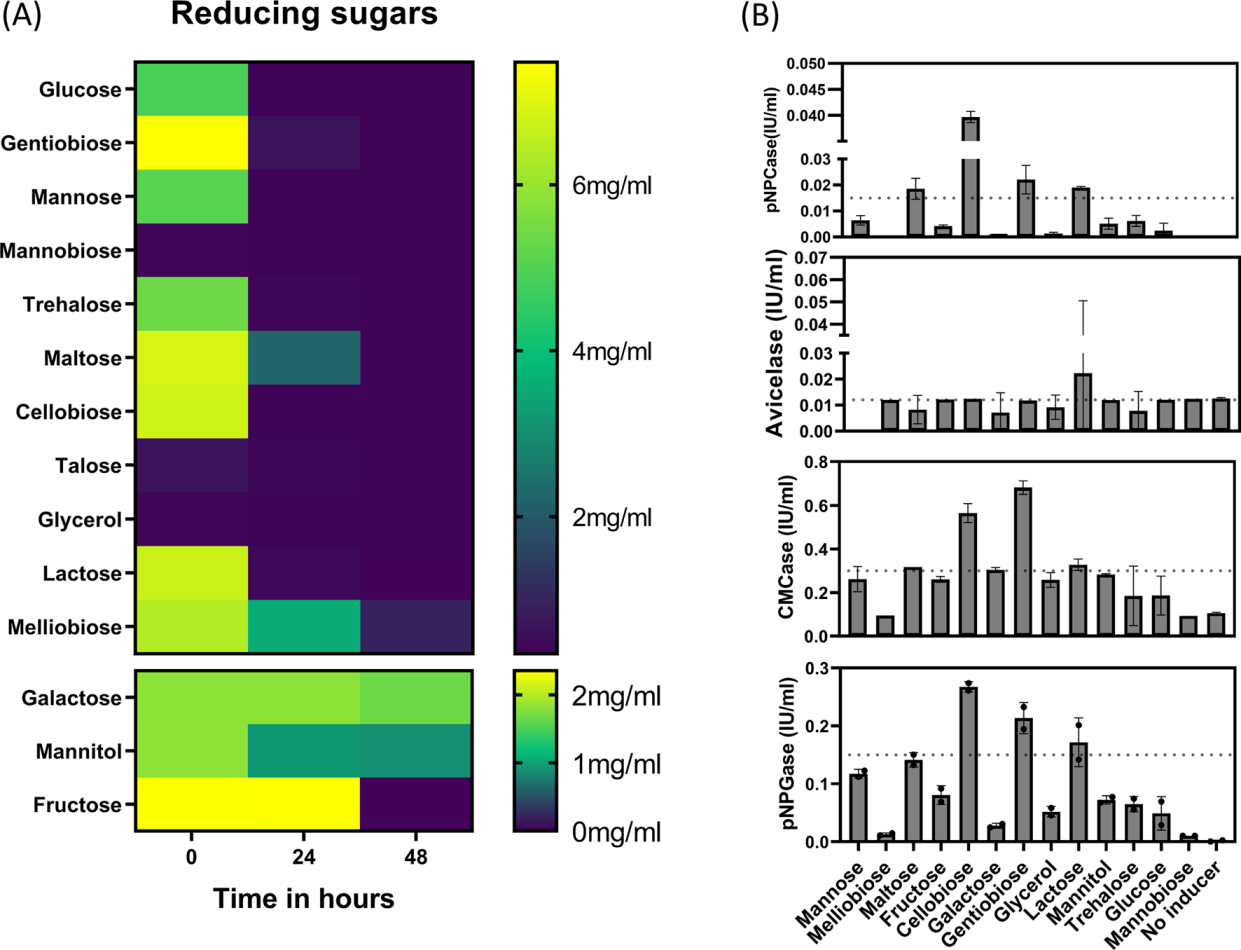
In vitro induction assays. **a** Consumption of inducers over time. Detected as a measure of reducing sugars using DNS method and HPLC. **b** Inducing capability of dominant metabolites was analyzed using in vitro assays after 24 h. To 10 mM of all soluble saccharide, pre-grown active mycelia was added and *Talaromyces* sp. was allowed to grow for 72 h. The samples were withdrawn at different time intervals for determination of cellulase activity (No substrate is no inducer)

Maltose, melibiose, cellobiose, gentiobiose, fructose, galactose, mannitol, trehalose, lactose, and glucose were the primary saccharides found in GC–MS analysis. We investigated the function of these saccharides in triggering the production of cellulase in *Talaromyces* sp. For this, we first analyzed the utilization of these saccharides by *Talaromyces cellulolyticus* by measuring their consumption over time. As a result, all sugars except mannitol were found to be fully metabolized within 48 h (Fig. 4a), indicating the presence of the transporters or transceptors for all other saccharides in *Talaromyces* sp.

We performed in vitro induction studies using each saccharide as a potential inducer. For this, minimal media containing 1% avicel was inoculated with equivalent amounts of mycelia, and metabolites were added at 10 mM concentration unless specified, and no inducer was used as a negative control for the experiment. The culture was incubated at 150 rpm for 72 h at 28 °C. The supernatant was then scrutinized for cellulolytic activity, such as CMCase, avicelase, pNPGase, and pNPCase. The dry mycelial weight was estimated to compare growth in different substrates. No significant difference was observed in the fungal growth. Of all the metabolites, it was found that gentiobiose, cellobiose, maltose, and lactose were the most effective at inducing cellulase activity, with a substantial effect seen with gentiobiose and cellobiose (Fig. 4b).

### Plackett–Burman design to identify saccharide combinations

Recent research articles by Jourdier et al., 2013 and Pirayre et al., 2020 showed that the mixture of sugars or inducers optimally induces cellulase production by *T. reesei* RUT C-30 [49, 50]. This study indicated that a three-factor mixture design with lactose, xylose, and glucose optimally induced the beta-glucosidase production in *T. reesei*, suggesting the importance of inducer combination for optimal enzyme secretion. Plackett–Burman statistical design was therefore used to find the ideal inducer combination that can lead to increased cellulase induction in *Talaromyces* sp. For this, 14 experiments were designed with different combinations of four variables (cellobiose, lactose, gentiobiose, maltose) and the significant factors responsible for the induction of cellulases by *Talaromyces* sp. MTCC25456 were identified. The upper and lower levels of each variable were chosen according to the previous experimental results of *in vitro* induction. Table 2 shows the effects of these components and their significance. A wide disparity in the total hydrolytic activity was observed in the experimental runs indicating the importance of appropriate combinations for optimal induction.

**Table 2:** Plackett–Burman design and responses

S. no.	Pattern	Lactose (mM)	Maltose (mM)	Gentiobiose (mM)	Cellobiose (mM)	pNPCase (mU/mL)	pNPGase (IU/mL)	CMCase (mU/mL)	Avicelase (mU/mL)
1	−−+−	0	0	10	0	47.55489	0.474856	91.99802	12.82219
2	+−++	10	0	10	10	44.40144	0.399312	82.8558	14.21495
3	0	5	5	5	5	27.24804	0.292579	89.66485	11.67941
4	−+−−	0	10	0	0	20.80252	0.165402	79.85601	11.34015
5	++++	10	10	10	10	36.98563	0.318569	96.56913	15.16132
6	+−++	10	0	10	10	40.38165	0.361539	92.71226	14.28638
7	+−−+	10	0	0	10	45.85688	0.379213	90.7124	13.71499
8	++−−	10	10	0	0	29.08467	0.292579	87.90307	12.91147
9	−−+−	0	0	10	0	49.35687	0.509509	105.997	15.32202
10	−−+−	0	0	10	0	47.65885	0.610697	100.1879	15.03632
11	−+−−	0	10	0	0	29.74308	0.188966	54.23874	11.25087
12	−+++	0	10	10	10	40.41631	0.361886	93.33126	13.12574
13	+−−−	10	0	0	0	46.09945	0.746538	106.5208	14.21495
14	−−−+	0	0	0	10	37.67869	0.606192	79.57031	11.62585
15	+++−	10	10	10	0	29.11932	0.446094	113.7584	15.572
16	+−−−	10	0	0	0	29.63912	0.200748	53.85781	11.25087
17	++++	10	10	10	10	42.98065	0.288767	83.71288	14.89348
18	+−−+	10	0	0	10	50.2232	0.415252	132.3762	15.4113
19	−−−+	0	0	0	10	48.76776	0.42149	90.23624	12.9829
20	−+++	0	10	10	10	46.93113	0.348025	88.28399	14.76849
21	−+−+	0	10	0	10	45.78757	0.306441	81.14163	13.55428
22	++−−	10	10	0	0	12.7976	0.122431	60.52402	11.41158
23	0	5	5	5	5	46.93113	0.503965	108.4255	17.89327
24	−−+−	0	0	10	0	40.27769	0.559757	100.8069	15.35773
25	−+−+	0	10	0	10	47.34697	0.306441	77.14191	13.50072
26	+++−	10	10	10	0	34.90643	0.500846	114.3298	16.4648
27	−−−−	0	0	0	0	2.886747	0.042729	44.00136	11.12588
28	−−−−	0	0	0	0	3.060013	0.036838	46.90592	11.10803

The actual by-predicted model shows no evidence of lack of fit; the full model is significant, as indicated by its *P*-value of 0.0325–0.0044 (Additional File 1: Fig. S8). The effect summary report shows that cellobiose and gentiobiose are significant at 0.05, whereas lactose and maltose display negligible effects (Additional File 1: Fig. S9A). Furthermore, the effects test result and scaled estimate indicate a positive impact of cellobiose and gentiobiose, whereas lactose and maltose negatively impact the cellulase induction (Additional File 1: Fig. S9B). Moreover, the t-ratio indicated that cellobiose and gentiobiose positively affected cellulase activity set at their high levels.

**Fig. 5:**
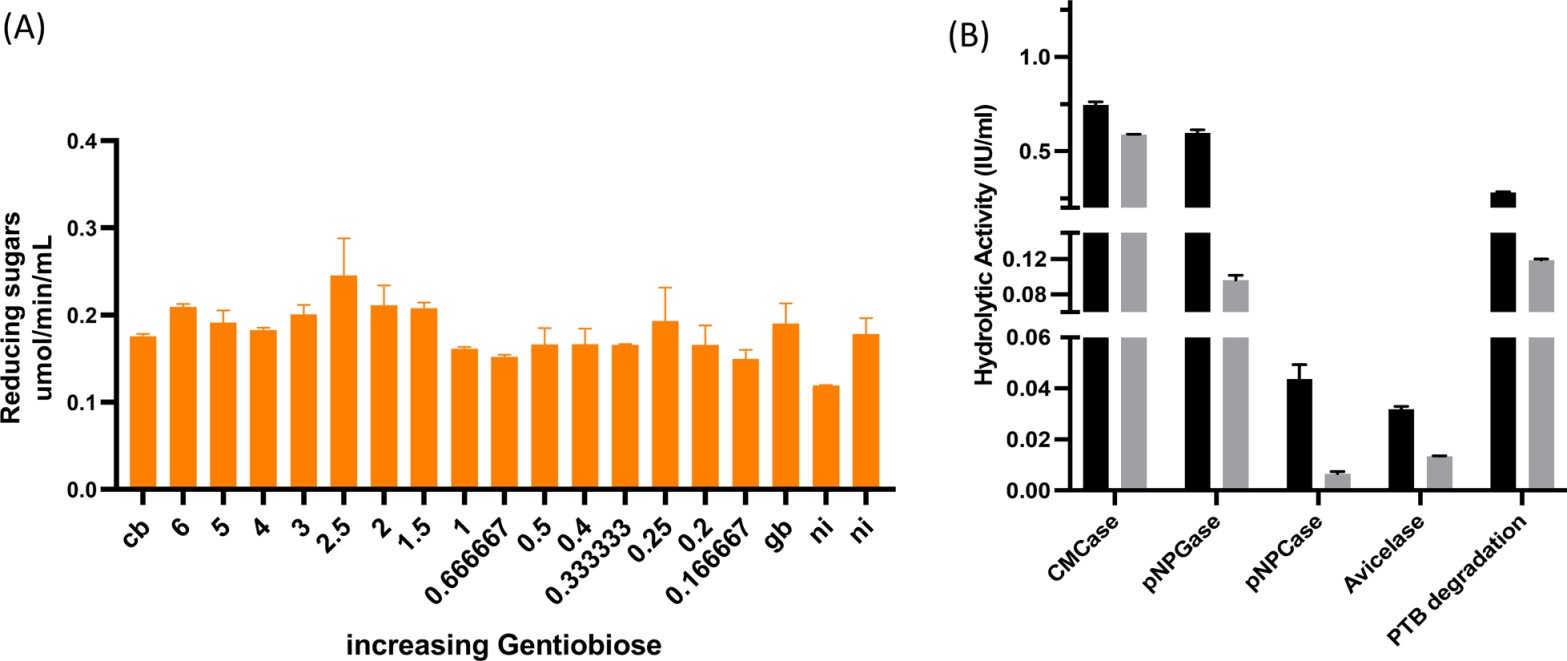
Stoichiometric balanced ratio of cellobiose and gentiobiose for optimal cellulase induction. **a** A range of saccharide combinations were analyzed for their effect on secretion of cellulase enzymes. The integrative effect was observed by evaluating the enzyme cocktail for its effect on hydrolysis of pre-treated wheat straw. The experiment was performed in two technical and two biological replicates. **b** A minimal culture medium containing 1% avicel. The supernatant was harvested after 48 h and different cellulolytic activity was estimated using standard biochemical assay (indicated with black bar). No inducer was user negative control (indicated with gray bar). The experiment was performed in two technical and two biological replicates

The Pareto chart also indicated that cellobiose displayed maximal effect, whereas maltose-mediated minimal/negative effect on cellulase induction. While it is clear from the experimental design that cellobiose and gentiobiose are two significant factors, the optimal levels of the individual factors were still unknown at this stage. Therefore, we designed another set of screening experiments with ratios of cellobiose and gentiobiose varying from 6:1 to 1:6, and the total concentration was fixed at 17.5 mM. Interestingly, supplementation of 2.5:1 (cellobiose: gentiobiose) to culture media displayed a significant increase in cellulase induction in *Talaromyces* sp. (Fig. 5a). It was observed that inducer enhanced CMCase, pNPGase, pNPCase, and avicelase activity by 1.6-, 6-, 7-, and 2.4-fold, respectively (Fig. 5b). Overall, stoichiometric balanced ratio (SBR) of 2.5:1 boosts the biomass hydrolysis by 2.4-fold.

### Submerged cultivation to evaluate effect of optimized inducer

**Fig. 6:**
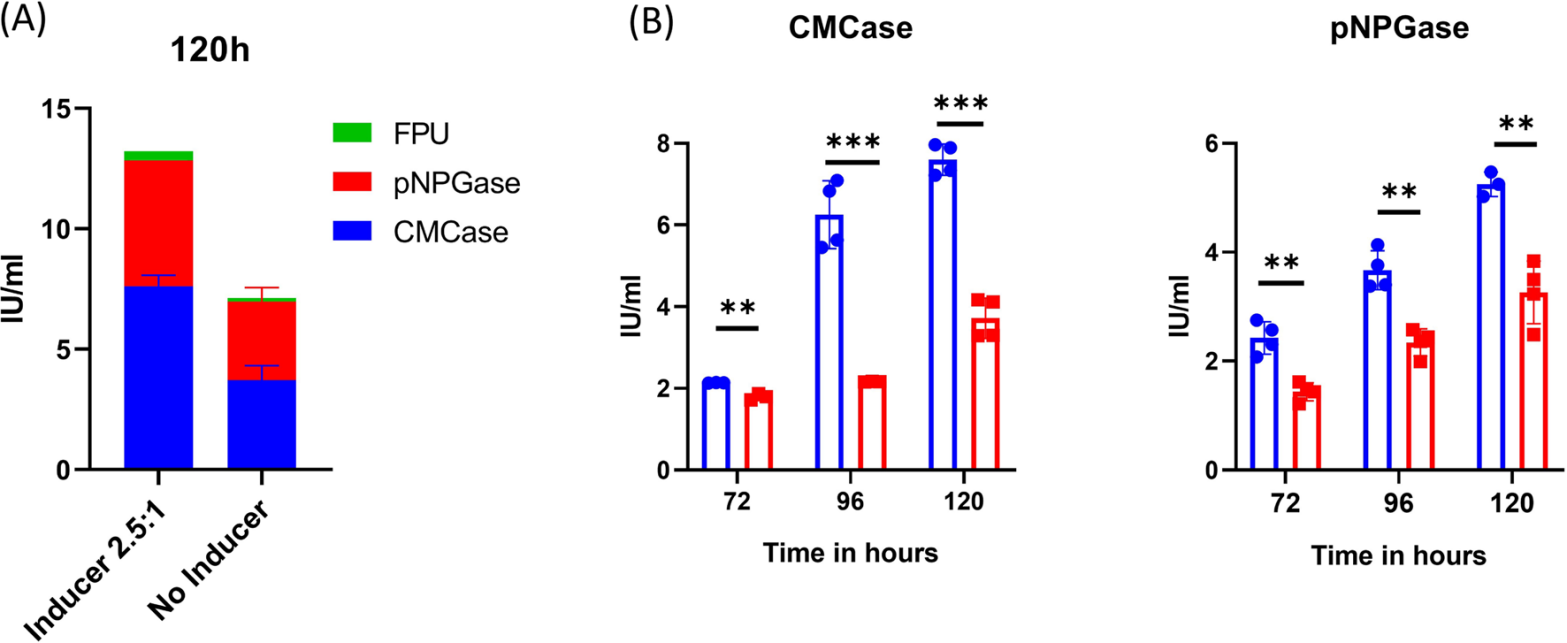
Submerged cultivation to evaluate effect of optimized inducer on cellulase induction. The inducers were added at the concentration of 12.5 mM cellobiose and 5 mM gentiobiose to complex culture medium containing 2.4%WB and 2.14% avicel. The supernatant was harvested after 72, 96, and 120 h and different cellulolytic activity was estimated using standard biochemical assay. No inducer was user negative control. The experiment was performed in two technical and two biological replicates. **a** Cumulative activities at 120 h. **b** Effect of inducer on time-dependent production of cellulases. The experiment was done in triplicate and standard deviation was calculated accordingly

To evaluate the effect of the optimized inducer on the biomass hydrolyzing capability of the strain, we compared the efficiency of the crude enzyme prepared using a stoichiometric balanced inducer ratio with that of uninduced crude enzyme. For this, the Talaromyces inoculum was raised for 36 h in PDB and used for inoculating the complex media containing 12.5 mM cellobiose and 5 mM gentiobiose, and no inducer was used as a negative control. As expected from our previous results, the activity of crude enzyme preparation in the presence of inducer outperformed that of the negative control (Fig. 6a). Precisely, it was perceived that there was a 175% increase in the cellulase production in the presence of inducer in terms of FPU.

According to Klein-Marcuschamer et al., 2011, the saccharification of complex biomass with the following conditions: 20% solids loading; 20 mg (0.5 FPU/mg) enzyme cocktail per gram of polysaccharide led to the development of an effective techno-economic model for inexpensive biofuel production [51]. We performed saccharification studies on pretreated wheat straw at 0.5FPU/g and compared with a commercial enzyme cocktail (cTec3). It was observed that crude enzyme preparation from our study hydrolyzed 65–76% of the total sugars from pretreated wheat straw, whereas only 55–68% hydrolysis was observed with commercial cellulase preparation after 12 h (Table 3).

**Table 3:** Comparison of Ctec3 and induced secretome from *Talaromyces* in terms of % saccharification

	Enzyme loading	2 h			12 h		
		No Inducer	Inducer	cTec3	No Inducer	Inducer	cTec3
Alkali treated wheat Straw	50 FPU/g	40.82	45.59	37.90	68.23	76.75	68.11
	100 FPU/g	46.12	50.13	35.72	52.35	65.21	55.49

### Genetic engineering to enhance cellulase production

CreA, a transcription factor, plays a major role in carbon catabolite repression in cellulolytic fungi and thus represses the expression of various cellulase genes. Several studies have shown that knockout of creA leads to increase in cellulase production [52, 53]. To further increase the cellulolytic potential of *Talaromyces* sp., we knocked out the CCR master transcription factor creA through *Agrobacterium*-mediated fungal transformation. (Additional File 1: Fig. S10, Lane 2).

**Fig. 7:**
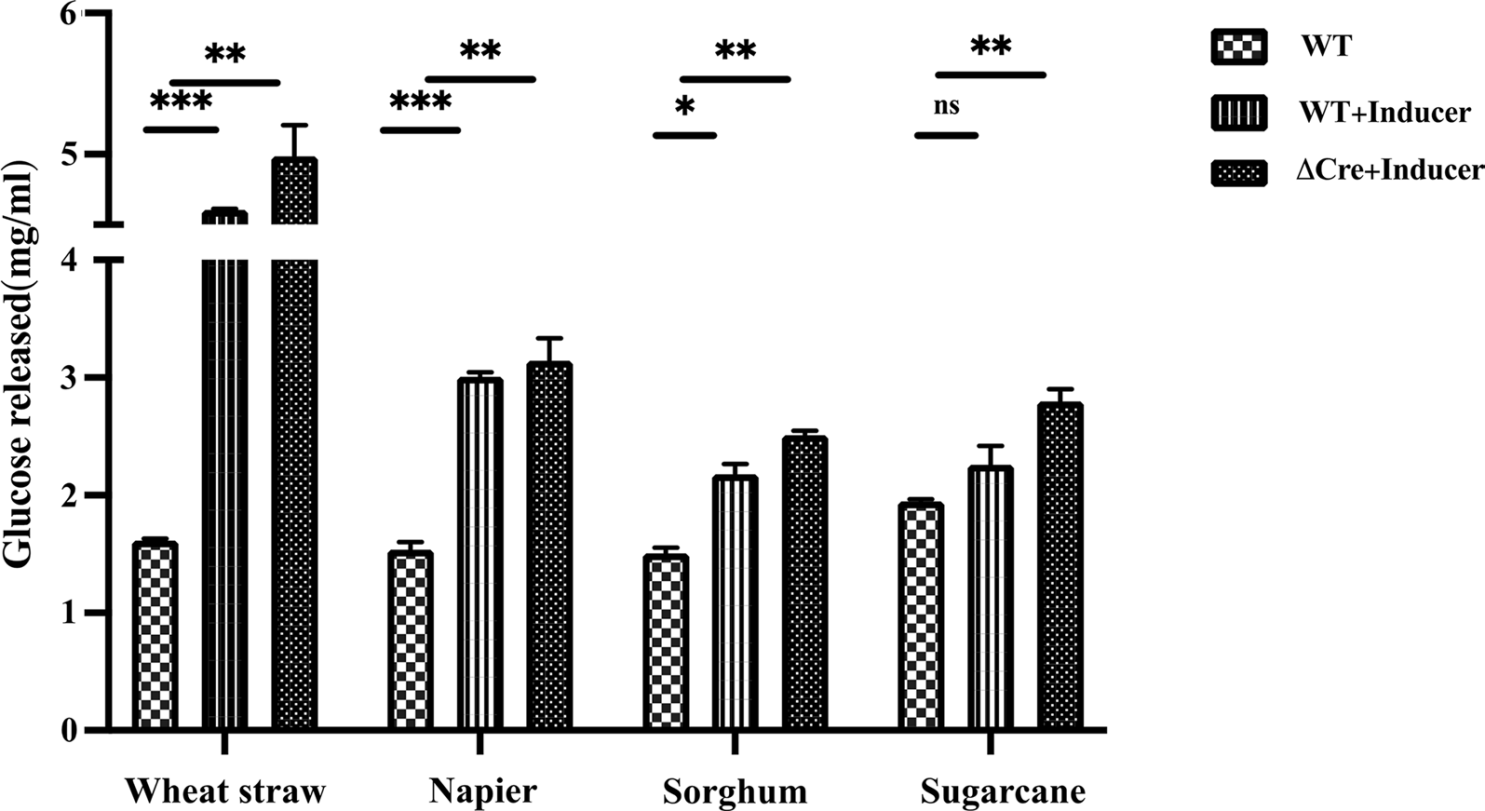
Combined effect of Inducers and cre knockout on composition of cellulase cocktail. The inducers were added at the concentration of 12.5 mM cellobiose and 5 mM gentiobiose to complex culture medium containing 2.4%WB and 2.14% avicel. Active mycelia were pre-grown in PDB for 36 h and used as inoculum. The supernatant were harvested after 5 days and concentrated using a 10 kDa centricon. No inducer was used as negative control. Total glucose released after 2 h of saccharification of pretreated wheat straw, Napier grass, sorghum, and sugarcane was estimated by the GOD–POD kit. The experiment was performed in two technical replicates

Different alkali pretreated biomass (kind gift from ICT, Mumbai) were used to test the saccharification efficiency of the ΔcreA strain (Additional File 1: Table S3). We discovered that the crude enzyme preparation from the creA strain demonstrated a statistically significant increase in the release of glucose from pretreated plant biomass when using the optimized inducer (Fig. 7).

## Discussion

In order to find effective biomass-hydrolyzing fungi from natural habitats and culture repositories, we compared their cellulolytic activities on standard substrates. We ranked them in accordance using the weighted sum model (WSM). The cumulative analysis determined that *Talaromyces cellulolyticus*, a novel fungal strain, had the highest level of cellulolytic activity. The strain was deposited in the MTCC stock center with the accession ID25456. Talaromyces secretome outperforms the commercial enzymatic mixture.

Genome and transcriptome analysis indicated rich diversity of CAZymes in *Talaromyces* sp. In addition, we identified the membrane-bound CAZymes after carefully examining the omics data together with biochemical investigation. The data were in accordance with a study performed by Kubicek et al., 1987, which predicted the presence of endoglucanase, glucosidase, and cellobiohydrolase on the surface of conidia. El-Gogary et al., 1989 also demonstrated basal levels of cellulases in uninduced fungi. This set of enzymes is believed as the first wave of attack on polysaccharide substrate [45]. Earlier, Vaheri et al. hypothesized the role of these membrane proteins in the synthesis of soluble inducer saccharides. To ascertain the role of membrane-bound CAZymes in generating inducing metabolites, we performed an in vitro reaction between complex biomass and resting spores. The supernatant was supplemented into fungal culture media, and its effect on cellulase secretion was evaluated, which revealed a 57% increase in total cellulase compared to the controls.

Further, the supernatant was scrutinized using GC–MS analysis, which predicted preponderance of the strain to produce carbohydrate derivatives, such as mannobiose, talose, gentiobiose, cellobiose, lactose, mannose, maltose, glycerol, and trehalose. We performed in vitro induction studies using each saccharide as a potential inducer. Of all the metabolites, it was revealed that gentiobiose, cellobiose, maltose, and lactose were the most effective at inducing cellulase activity, with a substantial effect seen with gentiobiose and cellobiose (Fig. 4b). Cellobiose, gentiobiose, and lactose are acknowledged to have roles in stimulating the production of cellulase [26, 27]; however, the rationale for selection of these saccharides was either based on random choice or a basic understanding of saccharides as a byproduct of cellulose degradation.

We examined these saccharides using Plackett–Burman and Fraction factorial designs to identify the optimal combination of inducers. As a result, we identified 2.5:1 (cellobiose: gentiobiose) as an optimal stoichiometric balanced ratio for inducing cellulase production in *Talaromyces* sp. As a result, the time required to produce cellulases was shortened by at least 24 h in the presence of the inducer, as evidenced by the fact that the pNPGase activity at 48 h in the presence of the inducer was comparable to that at 72 h in the absence of the inducer (Fig. 6b). Thus, we have shown that utilizing the inducers greatly reduces the fermentation time, which has a direct impact on the cost of lignocellulosic biofuel production [51].

To further augment the production of cellulases from *Talaromyces* sp., we developed a cre knockout strain. With newly identified inducers, the cre knockout displayed a 1.5- to 3-fold increase in biomass saccharification activities. Overall, this study develops a unique methodology for the rational selection of cellulase inducers, leading to increased cellulase production and biomass saccharification.

## Conclusion

In the present study, we identified the inducers for enhanced cellulase synthesis using the hypercellulolytic *Talaromyces* sp. as a model strain (Fig. 2a). Membrane-bound cellulases (Additional File 1: Table S2) facilitate avicel transglycosylation and degradation activity to produce soluble inducer metabolites. To investigate the byproducts of transglycosylation reaction, we used GC–MS-based metabolite analysis. Further, the positive correlation between transcriptome and metabolome allowed for the discovery of saccharides that may be essential in mediating cellulase induction. Interestingly, the cellulase system demonstrated improved cellulolytic activity toward saccharides, such as cellobiose and gentiobiose. These saccharides were examined using Plackett–Burman and Fraction factorial designs. The results revealed that the stoichiometric ratio of 2.5:1 (cellobiose:gentiobiose) was the most effective for stimulating cellulase synthesis and subsequent plant biomass saccharification. The efficiency of saccharification was further improved by creA knockout. More profound research into the underlying mechanisms may result in increased cellulase production.

## Material and methods

### Fungal growth conditions

*Talaromyces cellulolyticus* was isolated from a soil sample from Jawaharlal Nehru University (JNU), India using a standard serial dilution protocol and maintained on potato dextrose agar dishes. For transcriptomic studies, *Talaromyces* sp. was cultured in modified Mandel’s medium. Briefly, 5 × 10^6^ spores inoculated in 10 ml Mandel’s media containing 0.08% glycerol to prepare primary inoculum, which was then transferred to fresh 2-L erlenmeyer flask containing 500 ml media containing 0.4% glycerol and incubated at 28 °C for 24 h. The mycelia were then harvested and suspended in fresh media containing 1% avicel and incubated for 36 h at 28 °C [46].

Using similar protocol, induction studies were carried out with other mono and disaccharides. For in vitro induction studies, 10 mM sugar was used as inducer unless specified separately and supernatant samples were harvested after 36 h and 48 h of cultivation in 1% avicel for biochemical assays. All the experiments were performed in triplicate and no inducer was considered as negative control.

For submerged cultivation in complex media, mycelia raised in PDB for 36 h was transferred to a media containing 2.4% wheat bran and 2.14% avicel and supernatant samples were harvested after 120 h.

### Biochemical assays

The biomass disintegrating capability of *Talaromyces* sp. was analyzed using various biochemical assays. Endoglucanase/xylanase/avicelase assay: Briefly, 150 ul of appropriately diluted supernatant was added to 150 ul of 1%CMC/xylan/avicel solution (in 100 mM citrate phosphate buffer, pH 4.8), the reaction mix was incubated at 50 °C for 30 min, and release of reducing sugar was estimated using DNSA method [54]. pNPC/pNPG assay: Briefly, 5 mM *para*-nitrophenyl-d-glucopyranoside (pNPG)/ 0.1% para-nitrophenyl-d-cellobioside (pNPC) in 50 mM citrate buffer (pH 6.0)was incubated at 50 °C for 30 min. To the reaction mix 1% sodium carbonate is added to terminate the reaction and the absorbance was measured at 400 nm. Lytic polysaccharide monooxygenase activity: Here, reactions were performed using standard protocol by Ogunmolu et al. [55].

### Genome sequencing and pre-processing of the transcriptome raw data

The sequenced reads from the genome have been filtered and cleaned for obtaining the good quality data for generating an assembly. The first step in processing raw data include removal of the adapters using the Cutadapt-1.8.1 [56]. These reads without the adapters were processed further, using Sickle-1.210 (github.com/najoshi/sickle), to filter out the low-quality reads with a cut-off of Q20. Followed by that redundant reads were removed using FastUniq tool [57] to retain the optimum data for generating a good assembly.

The RNA sequence reads, from fungal mycelial samples collected at 36 h post-incubation with glycerol or avicel or pretreated biomass and at 24 h post-incubation with glucose, were also pre-processed using AdapterRemoval version 2.3.2 to trim the low-quality bases and adapters used in sequencing and get the Q20 quality raw reads. Further RNA-Seq reads were mapped on the assembled genome using HiSat2 [58] and kallisto and EdgeR were used for differential gene expression.

### Gene prediction and annotation

The assembled genome has been scanned for predicting the genes and their boundaries using various methodologies, like *ab initio* based, homology based, and evidence based. Augustus was used for the *ab initio*-based genes prediction utilizing the gene models from saccharomyces [59]. *Talaromyces cellulolyticus* protein sequences, fetched from NCBI, were used for homology-based prediction using exonerate [60]. Quality filtered raw reads of the transcriptome from the same organism were also used for gene prediction as the evidences. These reads were mapped on to the draft genome and generated various details, using tophat [61] and Cufflinks [62], to support the gene prediction. Ultimately, Evidence Modeler (https://github.com/EVidenceModeler) was used to get the consensus of the gene predictions from all the three methods and retrieved all the nucleotide and protein sequences of the predicted genes. *Talaromyces marneffei, Penicillium funiculosum, Talaromyces asperellum,* and *Talaromyces stipitatus* protein sequences were also used for annotation.

All the nucleotide sequence of the predicted genes were annotated with the Uniprot database using diamond blst [63] with e-value 0.003. Further, all the details, such as GO annotation, KEGG pathways, InterPro, RefSeq, eggNog, OrthoDB, and Pfam, were fetched using our in-house pipeline, named CANOPI.

CAZymes were predicted and compared for genomes of few closely related species, namely, *Botrytis cinerea, Aspergillus niger, Neurospora crassa, *and* Trichoderma reesei,* along with assembled genome in this study. This analysis was performed using the proteins of those species downloaded from NCBI and the predicted proteins from the genome study. The knowledge from CAZymes database (http://csbl.bmb.uga.edu/dbCAN/) has been used for predicting the CAZymes. Hidden Markov model algorithm was used in HMMER 3.0 (http://hmmer.org) was used to fetch the CAZYmes with e-value 10^–5^.

### Phylogenetics analysis

Domain sequence for beta-tubulin (BenA) gene and the nucleotide sequences for Interspace Transcribed Sequences (ITS2) of *Talaromyces cellulolyticus* were downloaded from NCBI. Homology search tools (blastp and blastn) were used with e-value 10^–5^. The identified genes/proteins were used for blast search, against NCBI NR database, to fetch the closely related sequences of various fungal species. Multiple sequence alignments have been generated, with the fetched set of sequences, for each gene using ClustalW [64]. Phylogenetic relationships were predicted using Maximum likelihood Mega7 with JTT model and 1000 bootstrap value using Maximum Likelihood algorithm.

### Metabolome analysis

To identify the inducer disaccharides produced by transglycosylation activity of constitutive cellulases on complex cellulose, 1 × 10^9^ spores of *Talaromyces* sp. were incubated with crystalline cellulose for 72 h at 50 °C. The supernatant was harvested and analyzed for in vitro induction experiment and GC–MS analysis. The supernatants from spore only, avicel only, and spore incubated with starch were used as negative control.

GC–MS analysis was carried out to scrutinize the saccharides present in the supernatant. Derivatization of lyophilized metabolite sample was carried out according to the method described previously [65]. Gas chromatography–mass spectrometry (GC–MS) analysis for untargeted analysis consisted of a Shimadzu Gas Chromatogram (GC-2010 plus) coupled with mass spectrometer (TQ 8050) and an auto sampler (AOC-20 s)–auto injector (AOC-20i). The injection volume was set at 0.2 μL and injection mode was set at Split Mode with split ratio of 5. Analysis was conducted using SH-Rxi-5Sil MS capillary column (30 m × 0.25 μm, 0.25 mm) (Restek Corporation, USA) and helium with flow rate of 1 ml min^−1^ as carrier gas. The oven temperature program was 80 °C isothermal heating for 2 min, a ramp rate of 5 °C min^−1^ to 250 °C, a 2 min withhold, and a final ramp of 10 °C min^−1^ with a 24 min withhold. Total run time for GC–MS was 67 min with solvent delay of 4.5 min. The chromatogram integration and mass spectra analysis were done through GC–MS solution software version 4.45 SP 1) and NIST14s and WILEY8 spectral library were used for derivatized metabolite identification.

### RNA extraction protocol

RNA was extracted from mycelia using TRIzol method as prescribed by Ambion Life Sciences. Briefly, the mycelia were filtered using a miracloth and crushed to a fine powder in liquid nitrogen. 1 mL of TRIzol reagent was added to 0.1 g of finely crushed mycelia and mixed well by vortexing. After 5 min of incubation 300 μl chloroform was added and invert mixed. After 3 min of incubation the mixture was centrifuged at 13,000 RPM for 15 min at 4 °C. The aqueous phase was transferred to a new centrifuge tube and chloroform extraction repeated. 500 μl isopropanol was added to the aqueous phase and stored at -20 °C overnight. Then, it was centrifuged at 13,000 RPM for 15 min at 4 °C. The pellet was washed in 300 μl of 70% ethanol and resuspended in 20–40 μl water. 1 μl of DNAse I (1 U/μl) with 4 μl 10× buffer was added to 1 μg RNA and incubated at 37 °C for 45 min. DNAse was denatured at 70 °C for 5 min.

### Transcriptome analysis

KEGG Pathway maps were used to identify genes involved in metabolism of different metabolites identified by GC–MS. The genes were identified in the genome of *Talaromyces* and confirmed using KEGG Ontology assigning them KO and EC numbers. Further their expression levels were correlated with the production of metabolites in a figure using PathVisio v3.3.

### Plackett–Burman design

After initial screening, a Plackett–Burman significant DOE (Design Of Experiment) was used to determine the extent of inducing capability of each inducer in a combination. Four biochemical assays (pNPCase, pNPGase, CMCase, and avicelase) were taken as responses in screening design with 4 identified metabolites as variable continuous factors and 1% avicel as a discrete 1-level factor. The design included 1 central point and no inducer as a negative control. All the runs were in biological replicates totaling to 28 randomized runs. JMP16 was used for analysis of the runs.

### Membrane protein extraction

Membrane proteins from spore were extracted using protocol as mentioned in Kubicek et al. 1988. Briefly, 10^8^ spores in a suspension were suspended in 0.1% Tween 80 and incubated at 4 ℃ with gentle shaking for 30 min. After centrifugation at 12000*g* for 5 min, the pellet was once washed in with Tween 80 and supernatant was finally resuspended in 20 mM sodium citrate buffer, pH 5.0.

### RNA extraction protocol

RNA was extracted from mycelia using TRIzol method as prescribed by Ambion Life Sciences. Briefly, the mycelia were filtered using a miracloth and crushed to a fine powder in liquid nitrogen. 1 mL of TRIzol reagent was added to 0.1 g of finely crushed mycelia and mixed well by vortexing. After 5 min of incubation 300 μl chloroform was added and invert mixed. After 3 min of incubation the mixture was centrifuged at 13,000 RPM for 15 min at 4 °C. The aqueous phase was transferred to a new centrifuge tube and chloroform extraction repeated. 500 μl isopropanol was added to the aqueous phase and stored at -20 °C overnight. Then, it was centrifuged at 13,000 RPM for 15 min at 4 °C. The pellet was washed in 300 μl of 70% ethanol and resuspended in 20–40 μl water. 1 μl of DNAse I (1 U/μl) with 4 μl 10× buffer was added to 1 μg RNA and incubated at 37 °C for 45 min. DNAse was denatured at 70 °C for 5 min.

### Cre knockout methodology

Using *Agrobacterium* mediated transformation, creA was knocked out following previously described protocols using homologous recombination [66, 67]. Briefly, *Agrobacterium* cells were transformed with pCAMBIA1302 containing deletion construct with 500 bp homologous regions. After 7 days of transfer to selection media, the transformant colonies were subsequently screened with a stringent selection criterion using minimal media plates containing 100 ug/ml hygromycin. After 3 rounds of screening, knockout was confirmed with PCR primers––Fwd 5′GTCTGTCTGTCGCTGCTAAACTC3′ and Rev 5′ACTTGGTTCTGGATGTATATTGCC3′.

### Biomass saccharification

Different biomass were used for this study, viz., alkali-treated wheat straw, alkali-treated sugarcane bagasse, alkali-treated Napier grass, and two-step treated sorghum. Biomass was treated with equal filter paper units of secretome (25 FPU/g, 3% solid loading, unless specified separately) at 50 ℃ for 2 h (in 100 mM citrate phosphate buffer, pH 4.8). The supernatant was collected by centrifugation and analyzed for total reducing sugars using DNS method and glucose release using GOD–POD kit from Megazyme.

## Supplementary Information

**Additional file 1. Fig. S1** Screening of hypercellulolytic fungi. **Fig. S2** Phylogenetic analysis of Talaromyces cellulolyticus MTCC25456. **Fig. S3** Synteny plot of assembled contigs of Talaromyces cellulolyticus MTCC25456. **Fig. S4** Representation of top 15 hits of Gene Ontology. **Fig. S5** Differentially expressed CAZYmes during cultivation on avicel and Pretreated biomass (PTB). **Fig. S6** Biochemical assay for membrane protein. **Fig. S7** Selection of optimum temperature for production of inducer molecules. **Fig. S8** Plackett Burman analysis. **Fig. S9** Effect summary of Plackett Burman design. **Fig. S10** CreA knock out. **Table S1** Assembly statics for contigs. **Table S2** Membrane Bound cellulases. **Table S3** Composition Analysis for different biomass. **Table S4** RT-PCR primers.

## Data Availability

The datasets generated and/or analyzed during the current study are available in the SRA repository wide SRP321539 and GenBank assembly wide accession number GCA_009805475.2.

## Abbreviations

AA: Auxiliary activity
CAZyme: Carbohydrate-active enzyme
CBM: Carbohydrate-binding module
CCR: Carbon catabolite repression
CE: Carbohydrate esterase
CMC: Carboxymethyl cellulose
dbCAN: DataBase for automated carbohydrate-active enzyme annotation
DEG/DET: Differentially expressed genes/transcripts
DNS: 3,5-Dinitrosalicylic acid
FDR: False discovery rate
FPU: Filter paper unit
GC–MS: Gas chromatography–mass spectrometry
GH: Glucosyl hydrolase
GOD–POD: Glucose oxidase–peroxidase
GT: Glucosyl transferase
KEGG: Kyoto encyclopedia of genes and genomes
MSTFA: *N*-Trimethylsilyl-*N*-methyl trifluoroacetamide
PL: Polysaccharide lyase
pNPC: Para-nitrophenol cellobioside
pNPG: Para-nitrophenyl glucopyranoside
PDB: Potato dextrose broth
