# Supplementary material for: Stoichiometric balance ratio of cellobiose and gentiobiose induces cellulase production in Talaromyces cellulolyticus
Source: Biotechnol Biofuels Bioprod. 2023 Mar 16;16:48. doi: 10.1186/s13068-023-02296-1 (PMC10018878; doi:10.1186/s13068-023-02296-1)
Supplement: Supplementary file 1 — Additional file 1. Fig. S1 Screening of hypercellulolytic fungi. Fig. S2 Phylogenetic analysis of Talaromyces cellulolyticus MTCC25456. Fig. S3 Synteny plot of assembled contigs of Talaromyces cellulolyticus MTCC25456. Fig. S4 Representation of top 15 hits of Gene Ontology. Fig. S5 Differentially expressed CAZYmes during cultivation on avicel and Pretreated biomass (PTB). Fig. S6 Biochemical assay for membrane protein. Fig. S7 Selection of optimum temperature for production of inducer molecules. Fig. S8 Plackett Burman analysis. Fig. S9 Effect summary of Plackett Burman design. Fig. S10 CreA knock out. Table S1 Assembly statics for contigs. Table S2 Membrane Bound cellulases. Table S3 Composition Analysis for different biomass. Table S4 RT-PCR primers. [file 13068_2023_2296_MOESM1_ESM.docx]

**Supporting Information**

**Stoichiometric balance ratio of cellobiose and gentiobiose induces cellulase production in *Talaromyces cellulolyticus***

**Authors & Affiliation:**

Shivam Aggarwal, Sathish Dorairaj and Nidhi Adlakha*

Synthetic Biology and Bioprocessing group, Regional Centre for Biotechnology, NCR-Biotech Science Cluster, Faridabad, India

*Any correspondence should be made to [**nidhi.adlakha@rcb.res.in**](mailto:nidhi.adlakha@rcb.res.in)

**Figures**

***
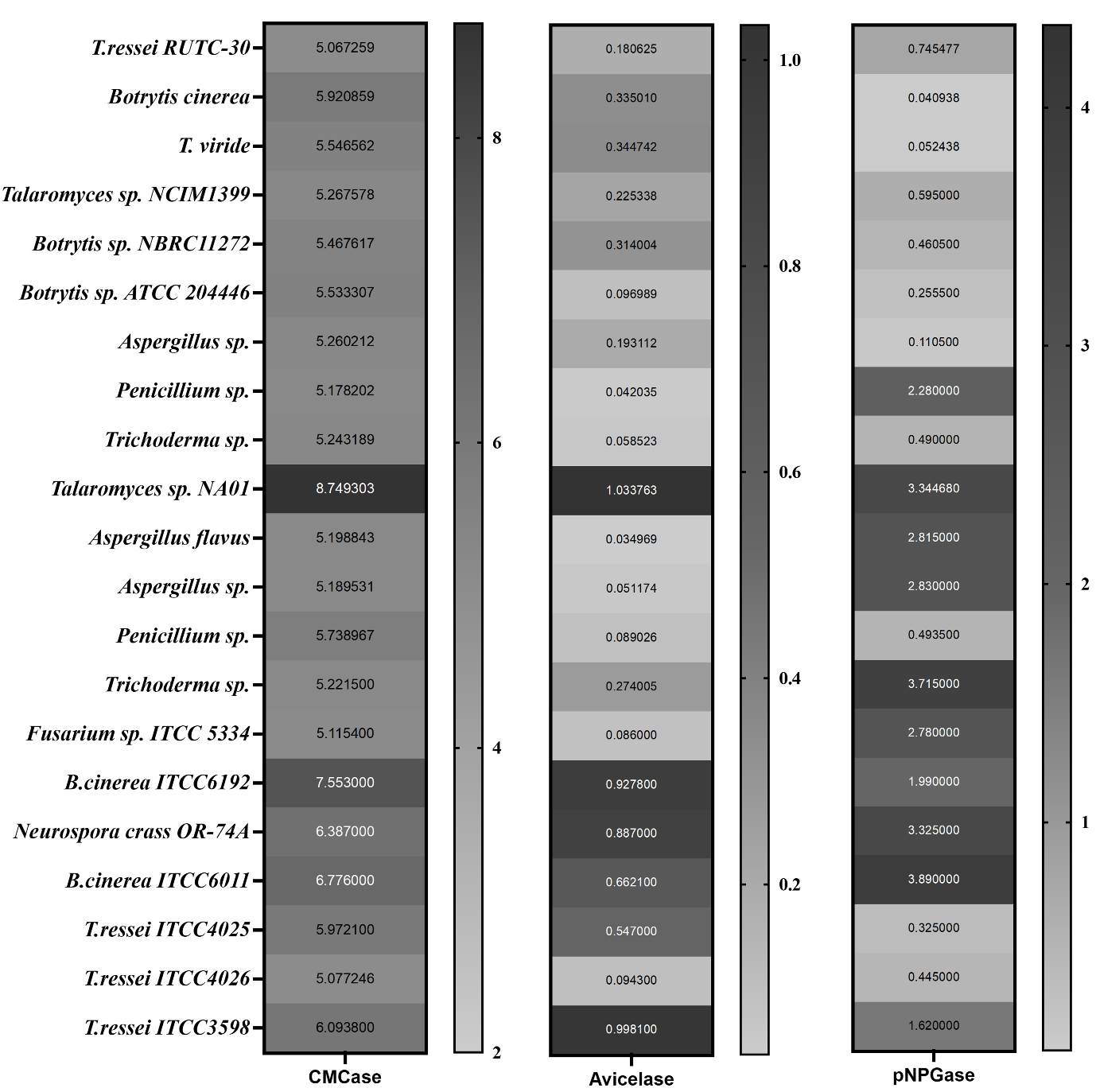
***

***Fig. S1 Screening of hypercellulolytic fungi***

*Various fungal strains with cellulolytic potential were collected from Culture collection centres such as ATCC, NCIM and ITCC and were used for comparison*

*
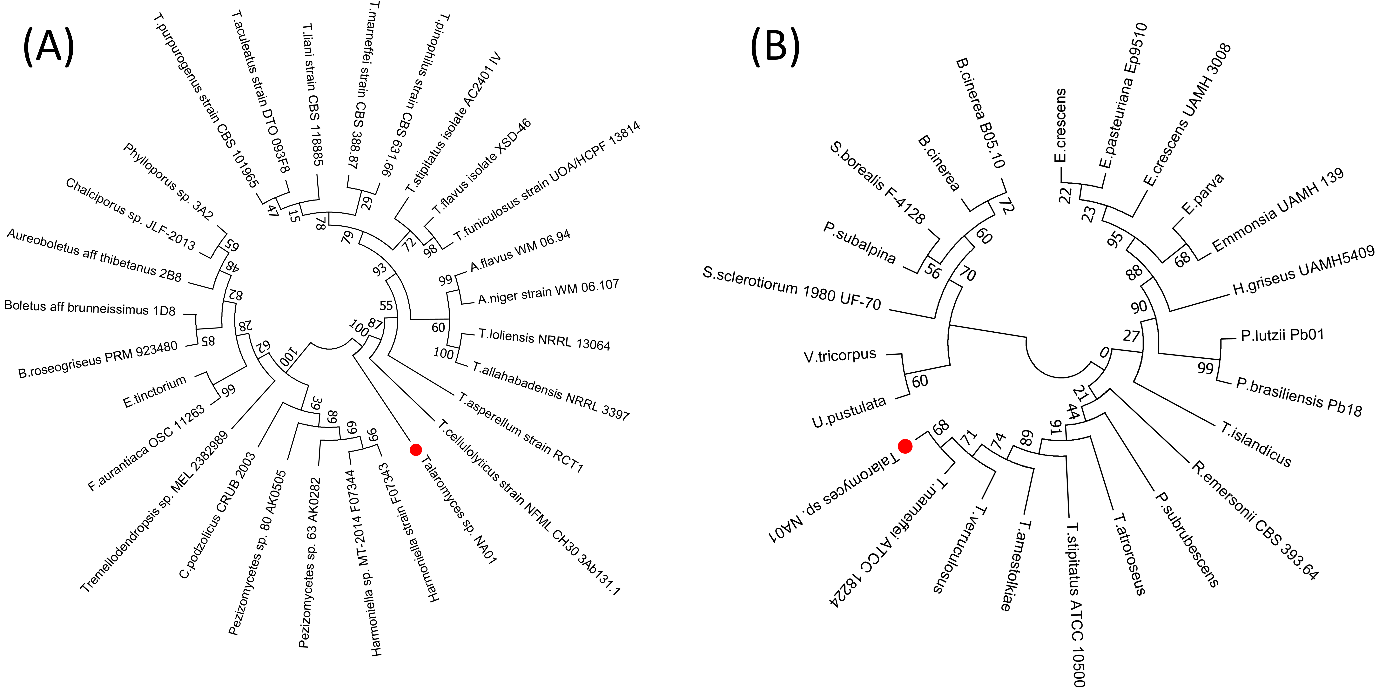
*

***Fig. S2 Phylogenetic analysis of Talaromyces cellulolyticus MTCC25456***

*Phylogenetic relationships of Talaromyces sp. NA01 were predicted using Maximum likelihood Mega7 with JTT model and 1000 bootstrap value using Maximum Likelihood algorithm. The phylogenetic analysis performed based on (A) Interspacer Transcribed Sequences (ITS2) and (B) beta tubulin (BT2) predicted that the isolate is closely related to Talaromyces cellulolyticus and Taloromyces marneffei ATCC 18224.*

*
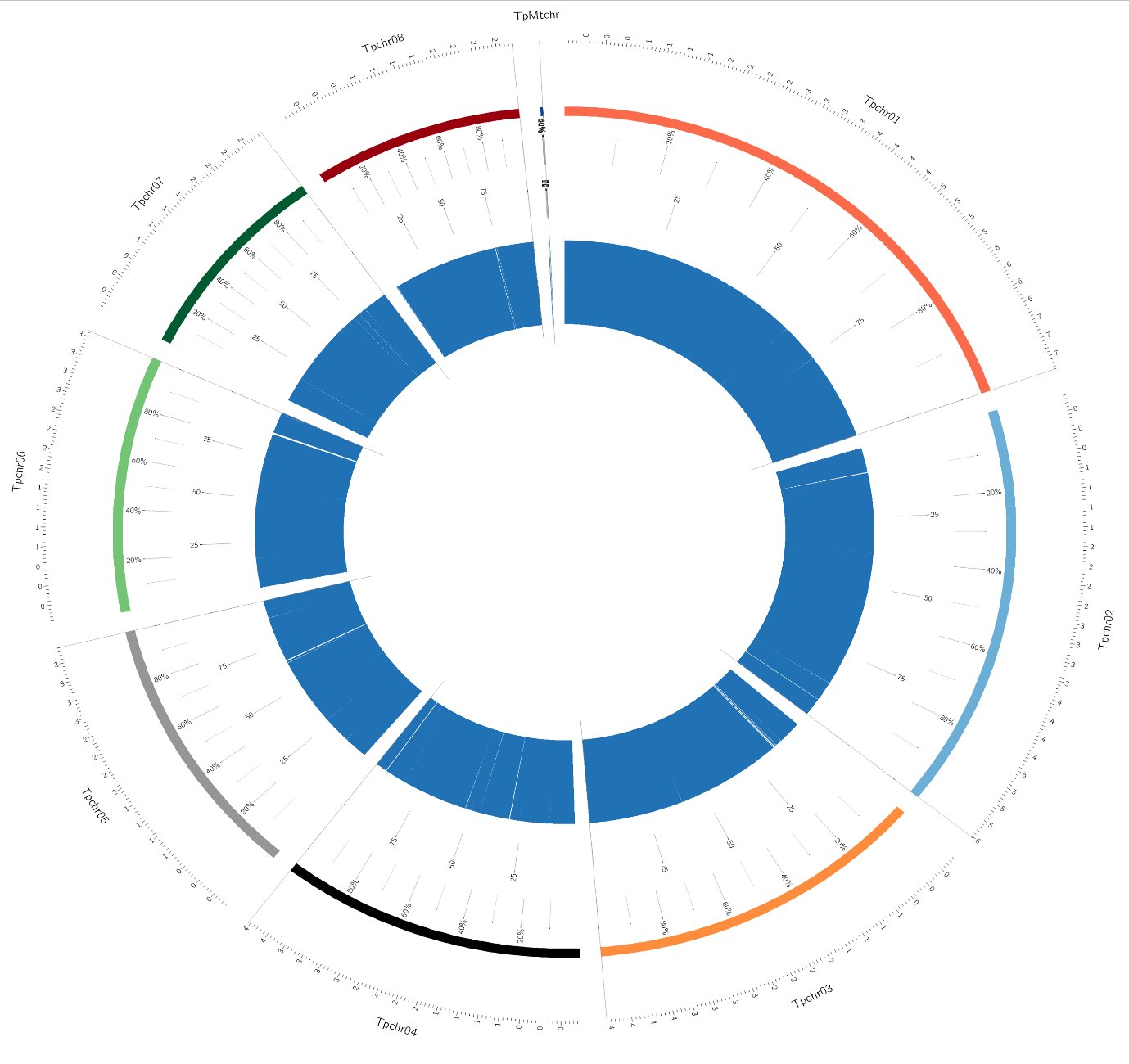
*

***Fig. S3 Synteny plot of assembled contigs of Talaromyces cellulolyticus MTCC25456*** (blue circle) with *Talaromyces* [*pinophilus*](https://www.ncbi.nlm.nih.gov/Taxonomy/Browser/wwwtax.cgi?mode=Info&id=128442&lvl=3&lin=f&keep=1&srchmode=1&unlock) Strain: 1-95 chromosomes (1–8).


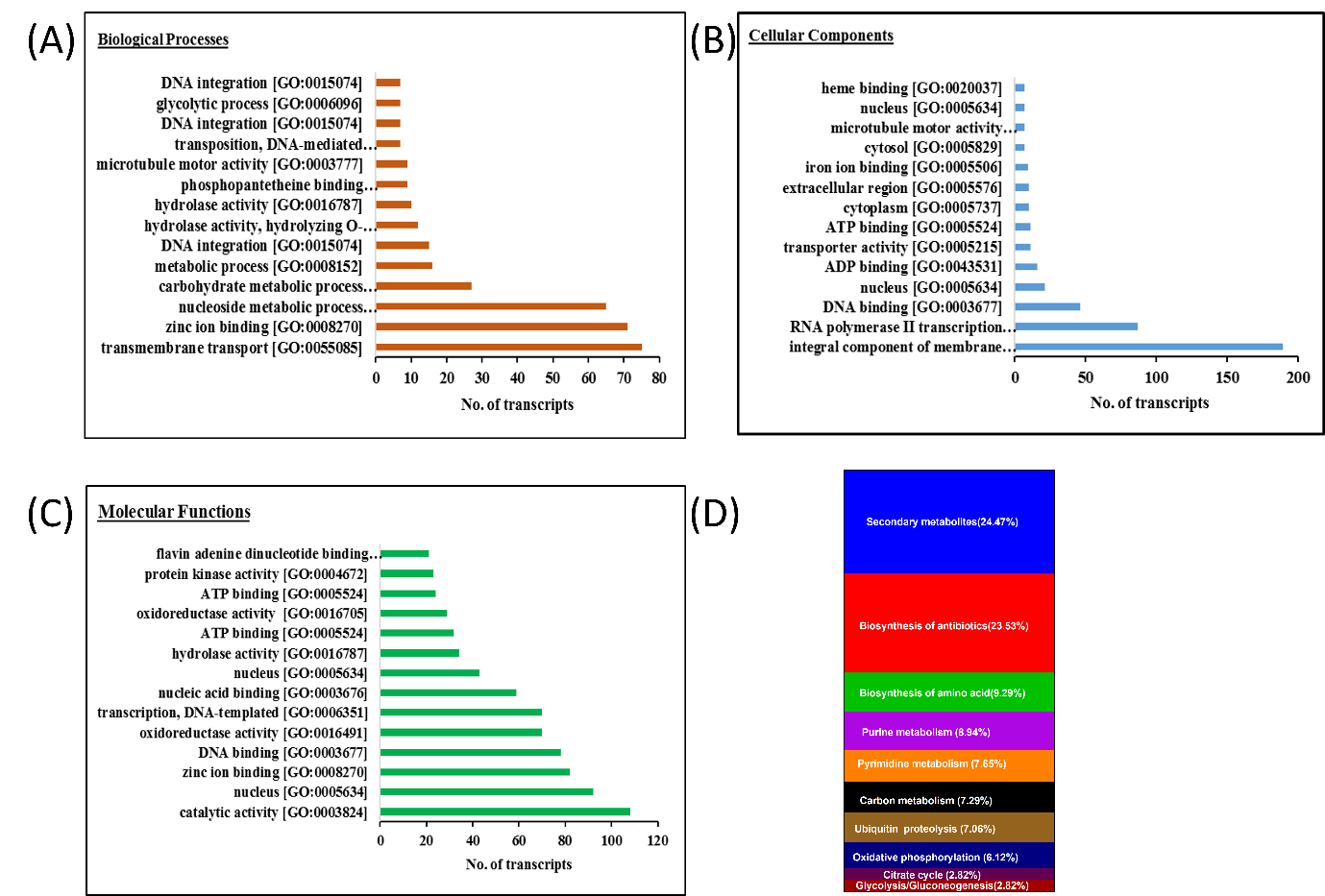


***Fig. S4 Representation of top 15 hits of Gene Ontology***

in(A) Biological (B) Cellular and (C) Molecular functions


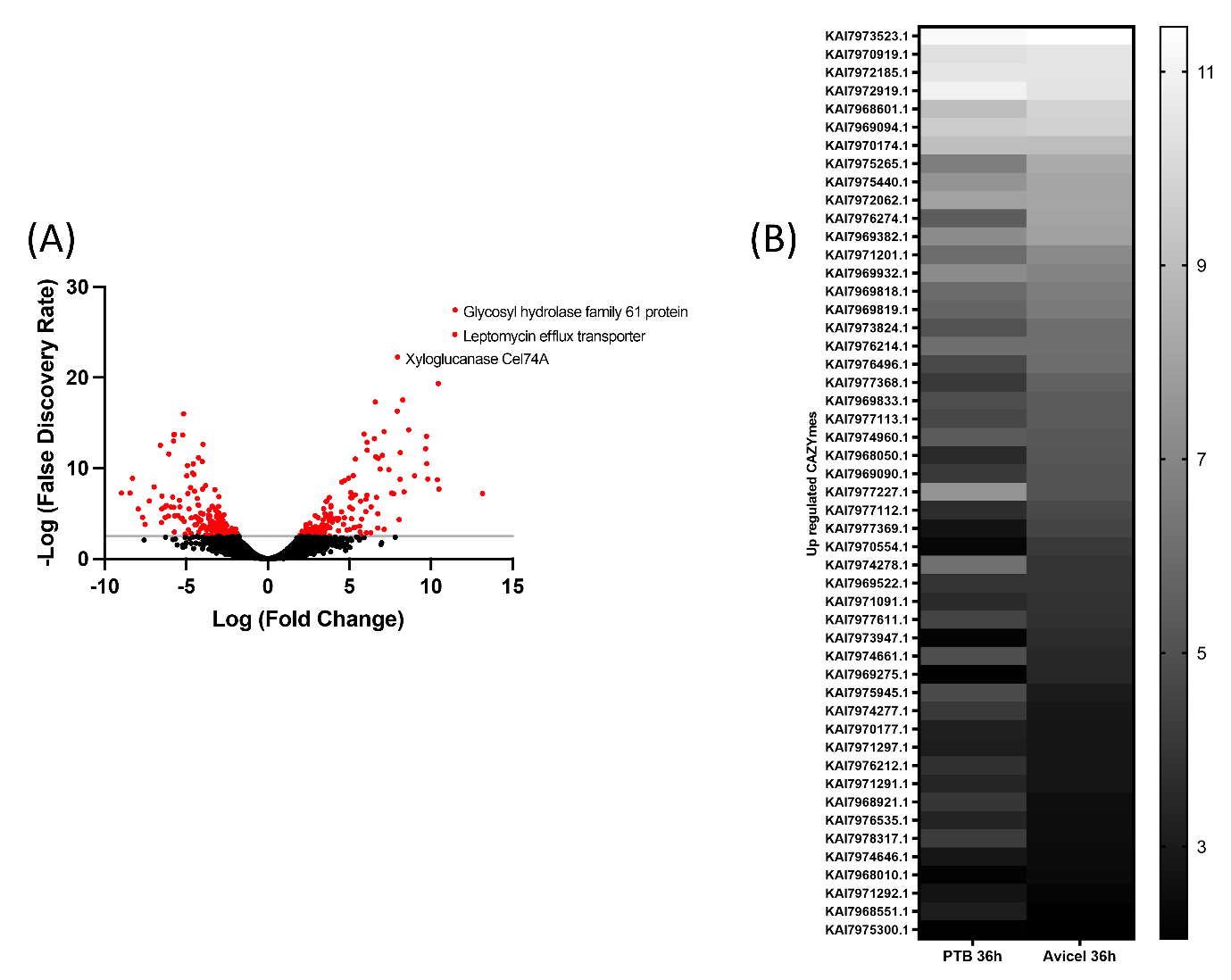


***Fig. S5 Differentially expressed CAZYmes during cultivation on avicel and Pretreated biomass (PTB).***

***(A)*** *Comparative transcriptome analysis of Talaromyces sp. in Avicel treatment and Glucose treatment. The volcano plot shows the differential gene expression in Avicel when compared with that in Glucose medium.* ***(B)*** *Heat map indicating common differentially upregulated CAZYmes 36h Avicel and 36h PTB induced compared to 24h Glc in Talaromyces sp. Shading is lighter for higher fold changes for all cases. Transcripts were considered significantly differentially expressed when fold change was at least two (p ≤ 0.05).*


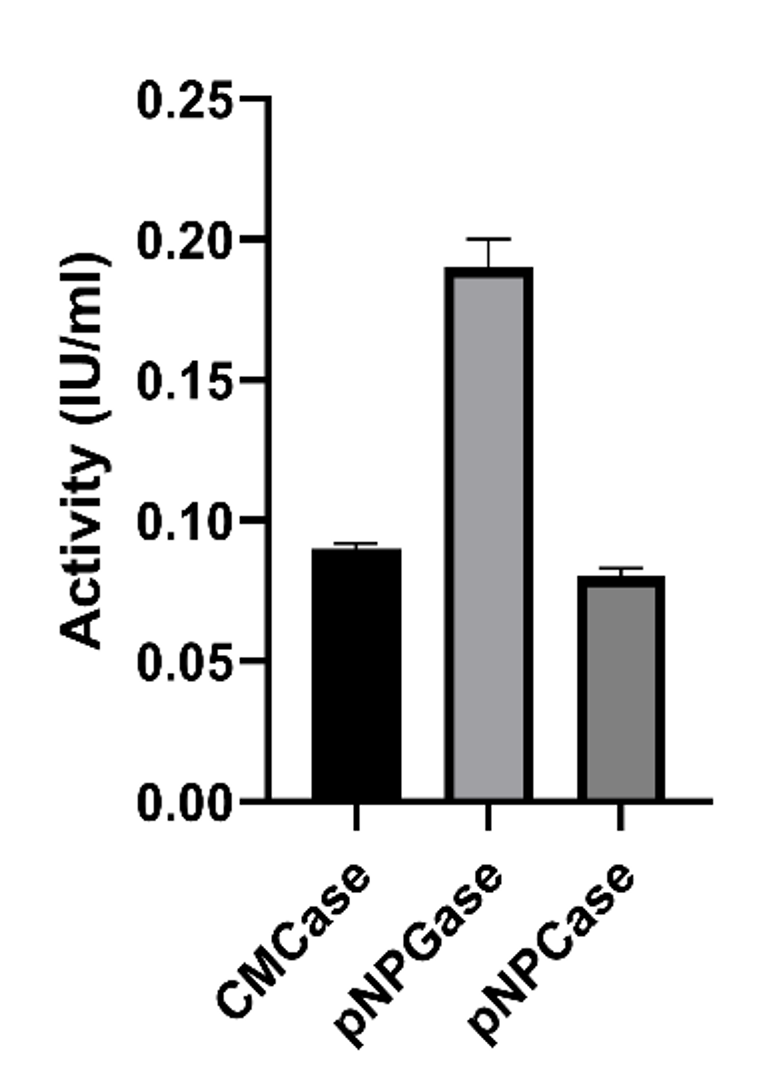


***Fig. S6 Biochemical assay for membrane protein.***

*The membrane protein was extracted according to standard protocol and assayed for endoglucanase and glucosidase type activity. The experiment was done in triplicate and standard deviation was calculated accordingly.*


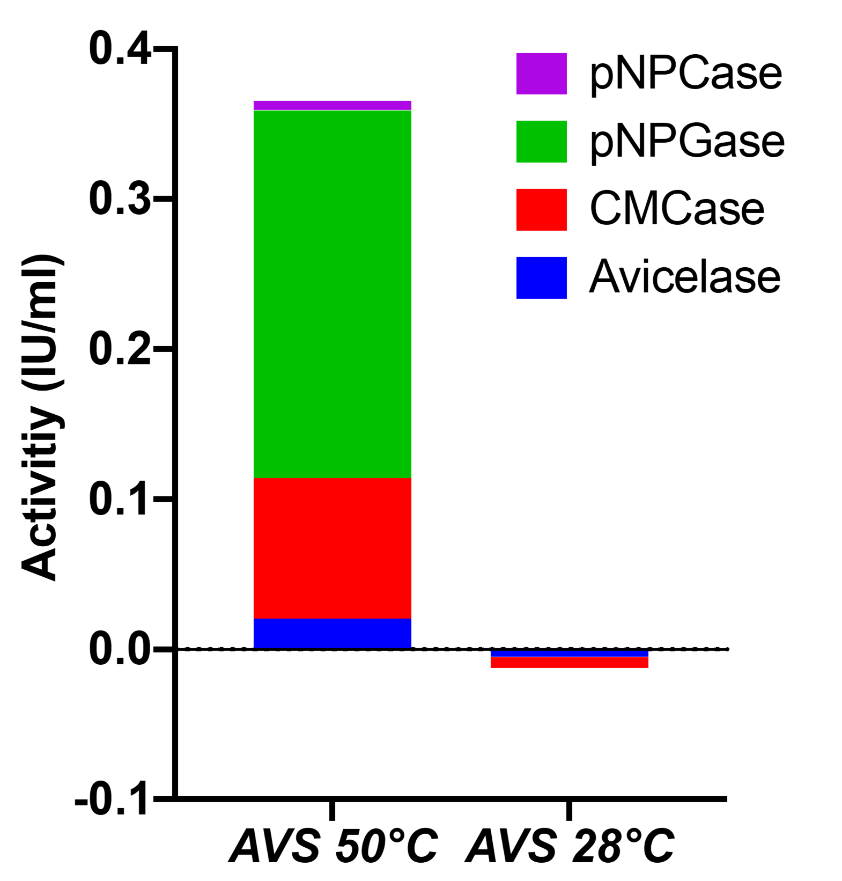


***Fig. S7 Selection of optimum temperature for production of inducer molecules.***

*The 1X10^9^spore were incubated with 1% avicel at 50ºC and 28ºC separately for 72h. The supernatant was supplemented in the growth media containing 1% avicel. The active Talaromyces mycelium were inoculated and supernatant was withdrawn after 36h for enzyme assays.*


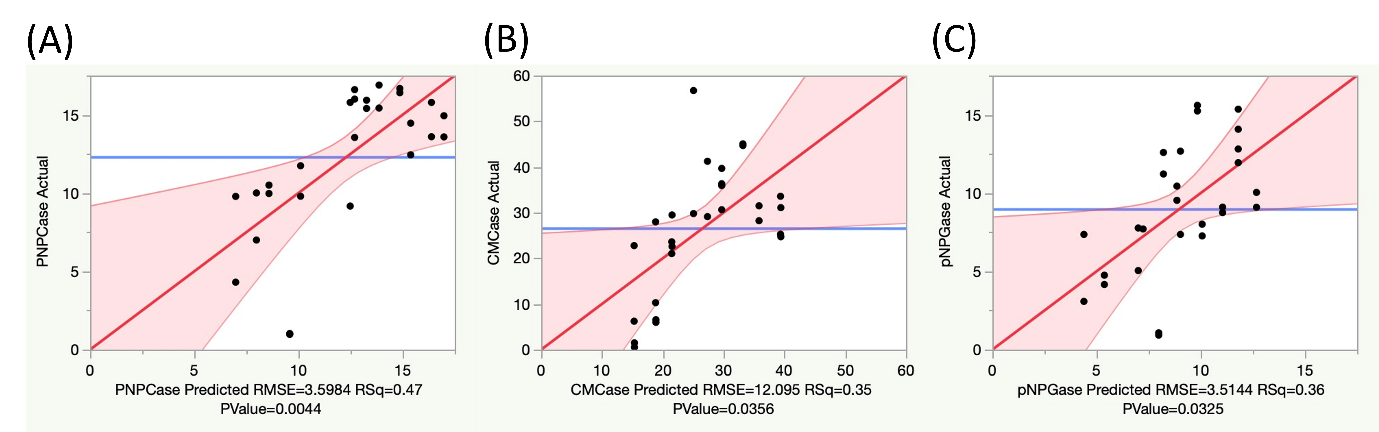


***Fig. S8 Plackett Burman analysis.***

*Actual by predicted model of pNPCase (A); CMCase (B); pNPGase (C) shows that the data is significant*

*
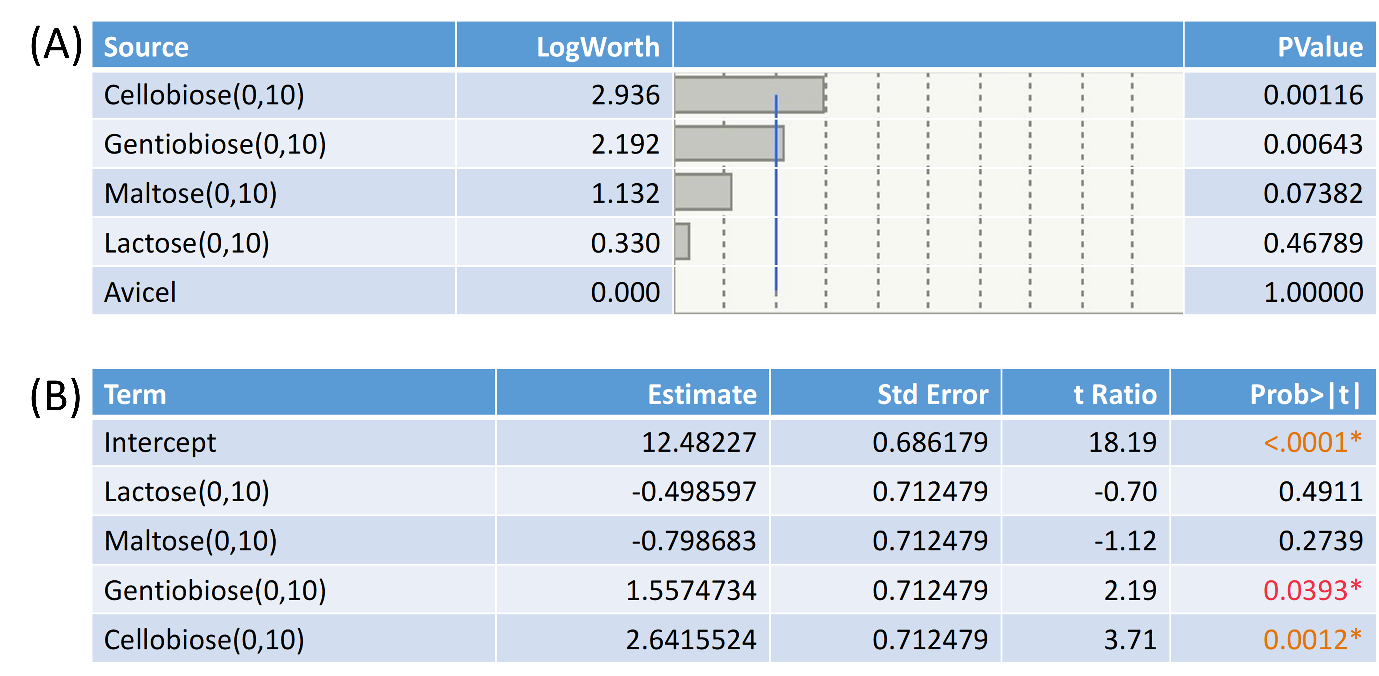
*

***Fig. S9 Effect summary*** ***of Plackett Burman design***

*(A) Effect summary report of Plackett Burman design.* *(B)Effect test result and scaled estimate results of Plackett Burman design*


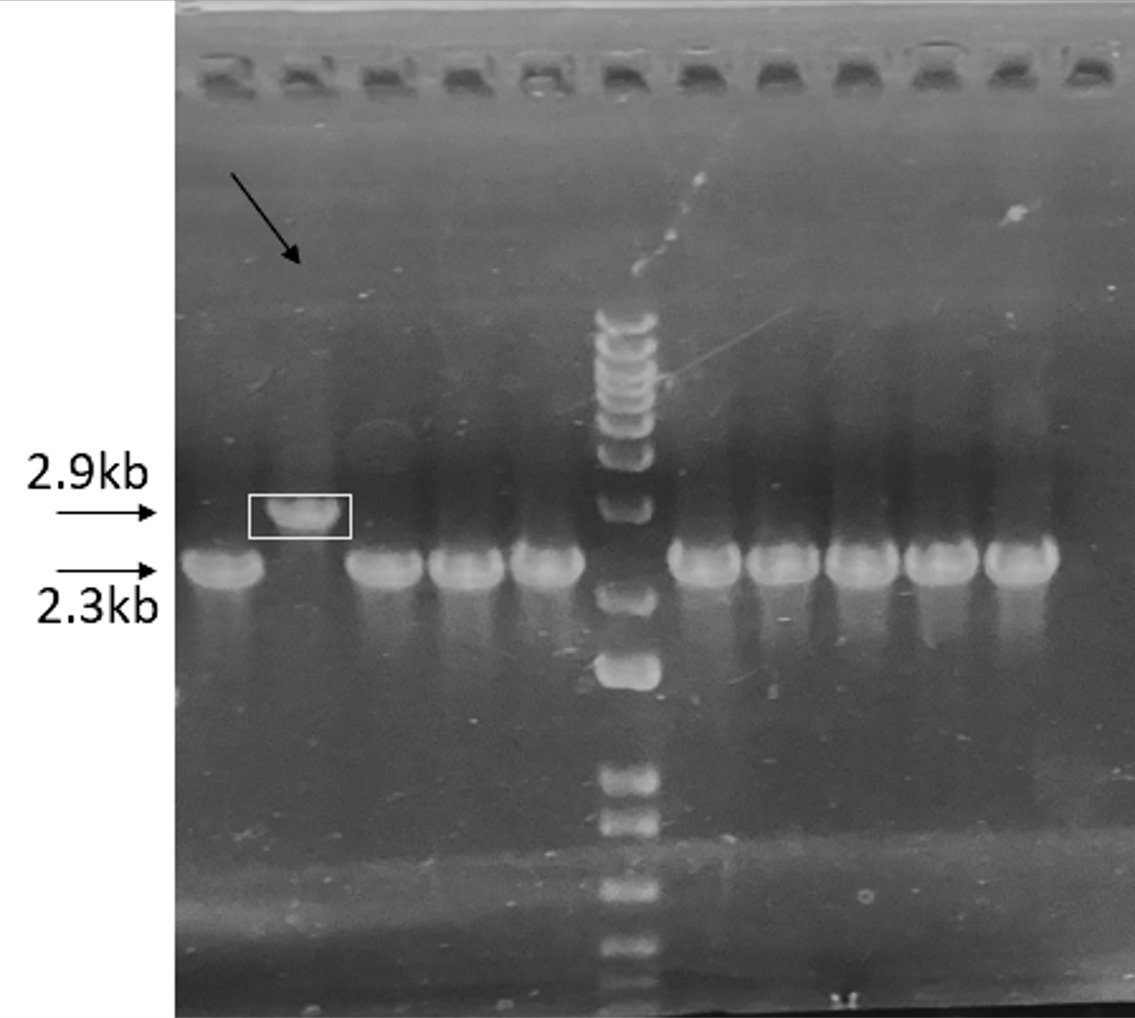


***Fig. S10 CreA knock out***

*1% Agarose gel. CreA knock out in Lane 2 shows a shift in the band. primers– Fwd 5’GTCTGTCTGTCGCTGCTAAACTC3’ and Rev 5’ACTTGGTTCTGGATGTATATTGCC3’*

**Tables**

**Table S1: Assembly statics for contigs**

| Assembly | MaSuRCa | Soap*denovo* k67 | Soapdenovo k78 | Spades |
| --- | --- | --- | --- | --- |
| # contigs (>= 0 bp) | 887 | 3,861 | 3,975 | 1,595 |
| # contigs (>= 1000 bp) | 676 | 1,164 | 1,250 | 577 |
| # contigs (>= 5000 bp) | 323 | 670 | 760 | 292 |
| # contigs (>= 10000 bp) | 230 | 502 | 583 | 213 |
| # contigs (>= 25000 bp) | 160 | 346 | 392 | 150 |
| # contigs (>= 50000 bp) | 116 | 219 | 227 | 112 |
| Total length (>= 0 bp) | 38,561,598 | 37,974,115 | 37,927,754 | 37,859,397 |
| Total length (>= 1000 bp) | 38,424,840 | 37,040,070 | 36,981,725 | 37,460,920 |
| Total length (>= 5000 bp) | 37,690,310 | 35,934,868 | 35,882,673 | 36,783,097 |
| Total length (>= 10000 bp) | 37,027,337 | 34,780,149 | 34,655,443 | 36,204,023 |
| Total length (>= 25000 bp) | 35,875,216 | 32,232,579 | 31,518,356 | 35,195,785 |
| Total length (>= 50000 bp) | 34,336,277 | 27,626,758 | 25,416,776 | 33,800,349 |
| # contigs | 828 | 1,664 | 1,736 | 830 |
| Largest contig | 1,633,654 | 394,282 | 371,764 | 2,804,526 |
| Total length | 38,535,829 | 37,389,315 | 37,317,952 | 37,646,110 |
| GC (%) | 47 | 47 | 47 | 47 |
| N50 | 391,761 | 112,808 | 84,868 | 382,360 |
| N75 | 192,766 | 48,035 | 40,989 | 194,981 |
| L50 | 30 | 101 | 124 | 26 |
| L75 | 66 | 228 | 284 | 60 |
| # N's per 100 kbp | 0 | 0 | 0 | 0 |
| Complete BUSCOs (C) | 1294 (98.4%) | 1288 (97.9%) | 1285 (97.7%) | 1294 (98.4%) |
| Fragmented BUSCOs (F) | 7 (0.5%) | 11 (0.8%) | 12 (0.9%) | 7 (0.5%) |
| Missing BUSCOs (M) | 14 (1.1%) | 16 (1.3%) | 18 (1.4%) | 14 (1.1%) |
| Total BUSCO groups searched | 1,315 | 1,315 | 1,315 | 1,315 |

**Table S2**: Membrane Bound cellulases

| **Protein NCBI ID** | **Transmembrane Helices** | **Putative Function** |
| --- | --- | --- |
| KAI7971606.1 | 15 | 1,3-beta-glucan synthase component FKS1 |
| KAI7970451.1 | 13 | alpha-1,4-glucan synthase |
| KAI7969028.1 | 12 | Vacuolar membrane protein |
| KAI7973501.1 | 11 | 1,4-alpha-glucan-branching enzyme |
| KAI7968906.1 | 10 | Oligosaccharyl transferase, STT3 subunit |
| KAI7971961.1 | 9 | Concanavalin A-like lectin/glucanases superfamily |
| KAI7970707.1 | 8 | Uncharacterized protein |
| KAI7977301.1 | 6 | Cellobiose dehydrogenase, cytochrome |
| KAI7971353.1 | 6 | Concanavalin A-like lectin/glucanase |
| KAI7977641.1 | 6 | Chitin synthase (EC 2.4.1.16) |
| KAI7978342.1 | 5 | Fungal chitin synthase |
| KAI7978312.1 | 5 | Cellobiose dehydrogenase, cytochrome |
| KAI7975560.1 | 4 | chitin synthase |
| KAI7971960.1 | 4 | putative glycosyltransferase YdaM |
| KAI7973107.1 | 4 | Glycosyl transferase, family 39 |
| KAI7977060.1 | 3 | Chitin Synthase |
| KAI7969626.1 | 3 | Cell wall acid trehalase |
| KAI7973529.1 | 3 | Fungal chitin synthase |
| KAI7974022.1 | 3 | D-lactate dehydrogenase |
| KAI7977256.1 | 3 | Mannan endo-1,6-alpha-mannosidase DCW1 |
| KAI7971355.1 | 3 | Glucans biosynthesis glucosyltransferase H |
| KAI7976887.1 | 3 | Cellulose synthase catalytic subunit [UDP-forming] |
| KAF3388267.1 | 3 | Chitin synthase G |
| KAI7973657.1 | 3 | Chitin synthase C |
| KAI7973455.1 | 2 | Glycoside hydrolase, family 2 |
| KAI7972919.1 | 2 | Beta-xylanase (EC 3.2.1.8) |
| KAI7978317.1 | 2 | Sterol esterase 2 |
| KAI7972733.1 | 2 | Glycosyl transferase, family 54 |
| KAI7976986.1 | 2 | putative glucan endo-1,3-beta-glucosidase btgC |
| KAI7974623.1 | 2 | Uncharacterized protein |
| KAI7977059.1 | 2 | Chitin synthase 6 |
| KAI7968742.1 | 2 | Glucosidase |
| KAI7971201.1 | 2 | Uncharacterized protein |
| KAI7976937.1 | 2 | Chitin synthase (EC 2.4.1.16) |
| KAI7977684.1 | 2 | Cellulose synthase catalytic subunit [UDP-forming] |

**Table S3: Composition Analysis for different biomass**

| **Composition** | **Cellulose (%)** | | **Hemicellulose (%)** | | **Lignin (%)** | | **Ash (%)** | |
| --- | --- | --- | --- | --- | --- | --- | --- | --- |
|  | **Raw biomass** | **Treated biomass** | **Raw biomass** | **Treated biomass** | **Raw biomass** | **Treated biomass** | **Raw biomass** | **Treated biomass** |
| **Sugarcane Bagasse** | 36.77 | 66.675 | 19.11 | 17.465 | 23.9 | 14.25 | 1.82 | 1.2 |
| **Wheat Straw** | 35.11 | 68 | 18.78 | 13 | 21.98 | 11.51 | 1.77 | 0.98 |
| **Napier Grass** | 22.34 | 59.01 | 18.21 | 20.98 | 29.37 | 18.64 | 3.32 | 0.62 |
| **Sorghum** | 31.59 | 74.61 | 20.78 | 13.5 | 27.35 | 10.52 | 1.92 | 0.67 |

**Table S4: Primers used for RT-PCR**

| Gene | Forward 5' to 3' | Reverse 5' to 3' |
| --- | --- | --- |
| egl1 | TGAACTGGCTGGATAGTGGTAG | CGAAGTTGACGACGATGATGAA |
| cbh1 | CTGATGCCTGCGGTGGTA | TGGTGGTGTCAACGGTCTT |
| cbh2 | GGCTCAACTCTTCGCAACAG | GGAGTCGCCAGATGTGTATGA |
| bgl | CTGGCAAAGTGGGATTGAAGTT | TGGCACCTGGAAGAGTATCG |
